# Hydrogel Microneedle Array‐Based Transdermal Dressing System for Multiplexed Assessment and Intelligent Therapy of Chronic Wounds

**DOI:** 10.1002/smll.202511542

**Published:** 2025-12-15

**Authors:** Md Sharifuzzaman, Gauri Hasabnis, Sheikh Ahmed Abu Saleh, Leonard Siebert, Jan‐Bernd Hövener, Gregor Maschkowitz, Zeynep Altintas

**Affiliations:** ^1^ Chair for Bioinspired Materials and Biosensor Technologies Institute of Materials Science Faculty of Engineering Kiel University Kaiserstr. 2 24143 Kiel Germany; ^2^ Chair for Functional Nanomaterials Institute of Materials Science Faculty of Engineering Kiel University Kaiserstr. 2 24143 Kiel Germany; ^3^ Section Biomedical Imaging Molecular Imaging North Competence Center (MOIN CC) Department of Radiology and Neuroradiology University Medical Center Schleswig‐Holstein and Kiel University 24118 Kiel Germany; ^4^ Institute for Infection Medicine Kiel University Brunswiker Str. 4 24105 Kiel Germany

**Keywords:** chronic wounds, conductive hydrogel‐forming microneedles array, correlation between wound exudate and interstitial fluid, diagnostics and therapy, laser‐scribed phase separation, transdermal dressing

## Abstract

Topical chronic wound dressings offer personalized management but have limited efficacy in sensing and delivering therapeutics due to their reliance on the wound surface. Herein, a theranostic hydrogel‐forming microneedles (HFMNs) dressing system is developed that penetrates the epidermis to continuously sample dermal wound interstitial fluid (ISF), providing high‐fidelity diagnostics and active therapy. The dressing is fabricated from a polyvinyl alcohol/chitosan hydrogel incorporating MXene for intrinsic antibacterial and pro‐healing properties. Central to the platform's design is a laser‐scribed phase separation process that converts a poly(3,4‐ethylenedioxythiophene): polystyrene sulfate/graphene oxide coating into highly conductive (384 S/m) and water‐stable electrodes directly on the HFMNs. This enables a multiplexed, replaceable array for continuously monitoring seven key wound biomarkers: glucose, uric acid, Na⁺, K⁺, Cl^−^, pH, and temperature. In vitro studies confirmed the dressing's cytocompatibility and antimicrobial efficacy against *E. coli* and *S. aureus*, while in vivo rat models showed accelerated wound closure. Critically, the HFMN system captured the biochemical dynamics of wound infection and healing from wound ISF with significantly greater fidelity and more distinct responses compared to conventional surface sensors sampling exudate. This work validates a robust platform that directly links deep‐tissue biomarkers to wound state, paving the way for personalized, proactive chronic wound management.

## Graphical abstract

This work develops a theranostic wound microneedle dressing integrates a therapeutic MXene‐infused hydrogel with a high‐conductive sensor array enabled by a novel laser‐scribing technique. The platform actively promotes wound healing and provides antibacterial action while continuously monitoring seven key biomarkers. The microneedle system captures the biochemical dynamics of wound infection and healing with significantly greater fidelity than conventional patches.

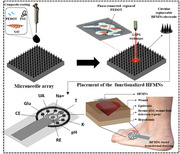

## 1 Introduction

Over 2 million people in Europe and 6.5 million people in the United States suffer from chronic nonhealing wounds (CWO), such as diabetic ulcers, nonhealing surgical wounds, burns, and venous‐related ulcerations,^[^ ^1^ ^]^ costing the health care system over $25 billion annually. Hemostasis, inflammation, proliferation, and remodeling are the four interdependent and contiguous phases of CWOs healing.^[^ ^2^ ^]^ In general, wound exudate production is at its maximum during the inflammatory phase and declines as the lesion heals.^[^ ^3^ ^]^ At each stage, the chemical composition (e.g., pH, glucose (Glu), uric acid (UA), and ions) and physical parameters (e.g., temperature (T)) of the wound environment change significantly, indicating the stage of wound healing and even the presence of infection.^[^ ^4^, ^5^, ^6^, ^7^, ^8^ ^]^ Planimetry is presently utilized in the clinical evaluation of wounds to qualitatively score characteristics such as slough reduction, granulation tissue formation, and reepithelialization.^[^ ^9^ ^]^ Currently, quantitative profiling of biochemical parameters is typically limited to laboratory testing, such as enzyme‐linked immunosorbent assays (ELISAs).^[^ ^10^ ^]^

Furthermore, several of the existing treatments, such as skin replacements, tissue transplants, mechanical wound psychotherapy, and others, can be helpful but often need surgery.^[^ ^1^ ^]^ Bacterial infection at the lesion site may result in tissue necrosis, by greatly impeding the healing process. A growing number of patients are being prescribed therapeutics, including both topical and systemic treatments, for chronic nonhealing lesions. An examination of the wound environment, nevertheless, reveals that an eschar generally causes a division of viable cells beneath the epidermis from its outermost layer.^[^ ^2^ ^]^ This necessitates the diffusion of therapeutics via the eschar in order to reach viable cells following topical application. Consequently, the topical delivery of medications results in a reduced local bioavailability compared to initial expectations.^[^ ^11^ ^]^

To address these therapeutic challenges, microneedle (MN) technology has emerged as a promising platform for creating multifunctional dressings for efficient drug delivery in CWO therapy.^[^ ^12^, ^13^, ^14^ ^]^ A key area of research is extending this capability to include diagnostics by accessing the dermal interstitial fluid (ISF). This ISF is the direct source from which wound exudate originates, when it leaks into a wound cavity, it forms the basis of the exudate that is typically sampled superficially.^[^ ^15^ ^]^ Accessing the ISF presents the possibility of obtaining a more immediate and accurate profile of the wound's biochemical state. To validate sensor performance in a clinically relevant environment, testing could be conducted in a high‐fidelity simulated interstitial wound fluid (SIWF). The formulation of this SIWF aims to include the key electrolytes, proteins, and metabolites that are believed to be present in both native ISF and clinical wound exudate.^[^ ^16^, ^17^ ^]^

Among the various MN architectures, which include solid‐coated^[^ ^18^ ^]^ and hollow MNs,^[^ ^19^ ^]^ HFMNs are particularly well‐suited for chronic wound applications due to their biocompatibility and softness. Unlike rigid, solid MNs that can cause tissue irritation or hollow MNs that offer only limited, discontinuous fluid extraction, HFMNs are fabricated from swellable, biocompatible polymers.^[^ ^1^ ^]^ They absorb fluid from the wound bed into their porous matrix, enabling continuous, equilibrium‐based sampling while simultaneously maintaining a moist, pro‐healing environment.^[^ ^12^, ^13^ ^]^ However, the realization of HFMNs for real‐time sensing has been fundamentally limited by the immense challenge of imparting high electronic conductivity. While conventional solid MNs can be metallized via techniques like sputtering to create conductive patterns, these methods are fundamentally unsuitable for hydrogel platforms.^[^ ^20^ ^]^ The significant mechanical mismatch between rigid metals and soft, hydrated polymer networks, combined with the aqueous nature of the hydrogel surface, leads to severe adhesion instability and makes direct metallization a formidable challenge.

Therefore, creating conductivity by integrating intrinsically conductive materials—such as conductive polymers and functional nanofillers—directly into the hydrogel matrix represents a more advanced and functionally superior strategy that enables a truly theranostic platform. Hydrogels incorporating conductive 2D nanofillers and polymers—such as polystyrene sulfonate, poly(3,4‐ethylenedioxythiophene):polystyrene sulfonate (PEDOT: PSS), graphene oxide (GO), and MXene—show significant promise.^[^ ^21^, ^22^ ^]^ On the contrary, the MXenes (Ti_3_C_2_T_x_), which are new 2D transition metal carbides, exhibit enhanced electronegative functional groups (─OH, ─F, ─O), significant metallic conductivity, and a greater specific surface area. 3D polymer hydrogel networks could be synthesized with enhanced mechanical stability through the incorporation of electronegative groups of Ti_3_C_2_T_x_.^[^ ^23^, ^24^ ^]^ In addition, MXene plays an important role in the healing of CWOs due to the antimicrobial activity of its surface functional groups.^[^ ^25^ ^]^ Conductive hydrogel‐based MNs composed of PEDOT: PSS have garnered the most attention due to their unique electrical and ionic dual conductivity and outstanding biocompatibility. However, when the PEDOT: PSS is mixed or embedded with the hydrogel, the electrical conductivity of the hydrogel decreases sharply (in the range of semiconductors).^[^ ^26^ ^]^ Moreover, PEDOT: PSS is unfavorable for long‐term operation in contact with biological tissues due to its relatively high Young's modulus (1–2 GPa), low stretchability (2% strain) due to the brittle PEDOT‐rich domain, and water instability due to the hydrophilic PSS‐rich domain.^[^ ^27^ ^]^ To convert PEDOT: PSS into water‐stable soft hydrogels, phase separation techniques that redistribute the networks between the hydrophobic and conductive PEDOT‐rich domain and the soft and hydrophilic PSS‐rich domain have been developed.^[^ ^28^, ^29^ ^]^ In addition, patterning and coating them with a high spatial resolution is a significant obstacle for applications involving MNs. Recently, laser‐scribed phase separation (LSPS) has emerged as a potential method for devising a novel biocompatible and ultrafast digital patterning process for the fabrication of water‐stable PEDOT: PSS hydrogel‐based electrodes, solving the dual challenges of conductivity and high‐resolution patterning simultaneously.^[^ ^30^ ^]^

Here, we introduce a high‐performance HFMNs‐based transdermal dressing system for the first time that uses a highly conductive, biocompatible, and minimally invasive replaceable hydrogel‐based MN array to monitor the physiological conditions of the transdermal wound fluid and perform intelligent therapy because of the hydrogel's antimicrobial properties. Compared to previously reported wearable sensors that rely on external wound bed exudate, theranostics at transdermal ISF can provide more comprehensive and customized information for effective chronic wound management. Building on a multifunctional polyvinyl alcohol (PVA) and chitosan (Ch) with MXene (Ti_3_C_2_T_x_) hydrogel foundation that provides intelligent therapy through its intrinsic antibacterial properties, we employ a pioneering technique—GO‐enhanced LSPS—to fabricate highly conductive (384 S/m) and water‐stable PEDOT: PSS electrodes directly onto the surface of the HFMNs array. This integrated approach yields a flexible, biocompatible dressing with a replaceable, multiplexed sensor array capable of continuously monitoring critical wound biomarkers in dermal ISF. By providing more comprehensive and customized information than systems relying on external wound exudate, and by establishing a potential correlation between these two biofluids, this work paves the way for a new generation of intelligent dressing systems for personalized and proactive chronic wound management.

## 2 Results and Discussion

### 2.1 Design and Fabrication of the Theranostic HFMN Dressing

The fabrication of the theranostic dressing is a multi‐stage, hybrid manufacturing strategy designed to integrate therapeutic functionality and high‐performance sensing capabilities into a soft, biocompatible platform. This process overcomes the limitations of single‐technique fabrication by combining distinct methods, each selected for its specific advantages in creating a complex, multi‐material device. The fabrication sequence comprises four key stages: 1) high‐fidelity mold generation using stereolithography 3D printing and soft lithography, 2) sequential in‐mold deposition of conductive and therapeutic layers to form the HFMN array, 3) post‐fabrication surface functionalization via LSPS to impart high, water‐stable conductivity, and 4) final assembly into a flexible, wearable dressing system. This approach balances precision, material compatibility, and scalability, enabling the creation of a sophisticated theranostic device without reliance on capital‐intensive cleanroom facilities.

#### 2.1.1 Fabrication of MN Array

**Figure 1:**
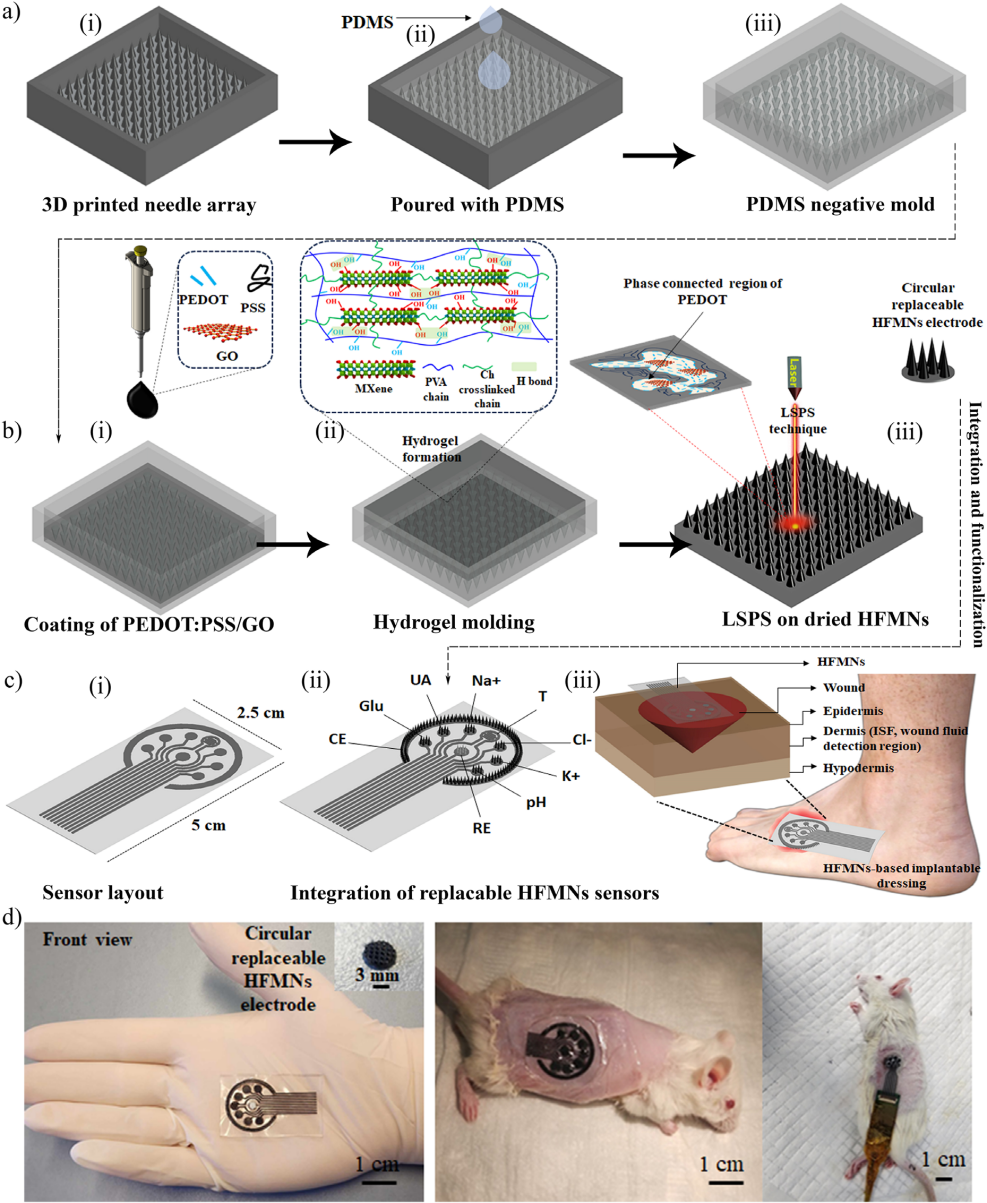
Schematic of the fabrication and assembly of the theranostic HFMN dressing system. a) Mold fabrication process, beginning with (i) computer‐aided design and stereolithography 3D printing of a master template, followed by (ii) casting of PDMS elastomer to create (iii) a high‐fidelity, flexible negative mold. b) Sequential fabrication of the conductive HFMNs within the mold. (i) A conductive PEDOT: PSS/GO ink is first coated into the MN cavities. (ii) The primary therapeutic hydrogel, composed of PVA, Ch, and MXene (Ti_3_C_2_T_x_), is then cast over the conductive layer and physically cross‐linked via a freeze‐thaw process (inset illustrates the resulting polymer network). (iii) The demolded array undergoes LSPS to create highly conductive, water‐stable electrode surfaces by reorganizing the PEDOT: PSS structure (inset illustrates phase separation). c) Integration into the wearable platform. (i) Fabrication of sensor layout on flexible substrate (CE‐counter electrode and RE‐reference electrode). (ii) These circular cut HFMNs are mounted onto a flexible polar array substrate with pre‐patterned contacts. (iii) The complete sensor array is integrated into a transparent, breathable medical adhesive dressing for application to CWOs. d) Photographs showing the flexible, fingertip‐sized HFMN dressing, its application to a wound on a rat model, and the complete in vivo measurement setup connected to a portable electrochemical workstation for real‐time data acquisition.

The fabrication process commences with the creation of a high‐resolution master mold. A solid MN array, serving as the master template, was designed using computer‐aided design (CAD) software and fabricated via cleanroom‐free stereolithography 3D printing (**Figure** **1**a,i). This technique was strategically chosen for its ability to rapidly produce complex microstructures with sufficient resolution for skin penetration at a significantly lower cost than conventional photolithography or two‐photon polymerization.^[^ ^31^ ^]^ The master array consists of 49 × 43 conically‐shaped needles, each 800 µm in height with an inter‐needle spacing of 700 µm, arranged on a 60 × 40 mm base. This positive master was then used to create a high‐fidelity negative mold via soft lithography (Figure 1a,ii,iii). A liquid polydimethylsiloxane (PDMS) elastomer was cast over the master and cured, yielding a flexible, non‐stick negative replica that can be used repeatedly, enhancing the scalability of the fabrication process.

To construct the conductive HFMNs, a sequential deposition process was employed within the PDMS negative mold, a critical sequence depicted in Figure 1b(i). First, a conductive composite ink was prepared by sonicating GO within an aqueous dispersion of PEDOT: PSS. This ink was then drop‐coated into the MN cavities of the PDMS mold, forming a thin, uniform electroactive lining. The inclusion of GO is critical not only as a conductive filler but also as a processing aid that enhances laser absorption, facilitating the subsequent LSPS treatment at lower laser powers (LP).

Following the deposition of the conductive layer, the primary hydrogel precursor solution was cast into the ink‐lined mold (Figure 1b,ii). This multifunctional hydrogel was formulated for optimal biocompatibility, mechanical integrity, and therapeutic activity. The biocompatible PVA can form a 3D network with Ch via cross‐linking by repeated freezing and thawing, and Ch can improve the porosity and water absorption capacity of the hydrogel—properties essential for efficient ISF sampling.^[^ ^32^ ^]^To impart both mechanical strength and therapeutic function, Ti_3_C_2_T_x_ MXene was incorporated as a nanofiller. MXene, synthesized via a minimally intensive layer delamination (MILD) method^[^ ^33^ ^]^ yielded several layers with an accordion‐like morphology, as confirmed by their subsequent physical properties (Figures S1 and S2, Supporting Information). The electronegative functional groups (─OH, ─F, ─O)^[^ ^23^ ^]^ on the surface of the MXene nanosheets form robust hydrogen bonds with the PVA and Ch polymer chains, creating a physically cross‐linked 3D network that significantly improves the hydrogel's mechanical stability (see Figure 1b,ii inset). Furthermore, MXene possesses intrinsic antibacterial properties, rendering the hydrogel actively therapeutic for preventing wound infections.

The solidification of the hydrogel within the mold was achieved through a physical cross‐linking method involving two repeated freeze‐thaw cycles. During this process, the freezing and subsequent thawing of the aqueous solution induce the formation of crystalline domains within the PVA chains, which act as physical cross‐links to stabilize the hydrogel network. This method was deliberately chosen over chemical cross‐linking to avoid the use of potentially cytotoxic reagents, ensuring the high biocompatibility required for a device in direct contact with a chronic wound. After the final thaw cycle, the fully formed HFMN array, now featuring an integrated conductive surface, was carefully peeled from the flexible PDMS mold and dried under vacuum in preparation for further modification.

#### 2.1.2 Laser‐Scribed Phase Separation (LSPS) Treatment

Although the in‐mold coating process integrates a conductive layer, the as‐deposited PEDOT: PSS/GO composite exhibits only moderate electrical conductivity and poor stability in aqueous environments due to the insulating and hydrophilic nature of the PSS component. To overcome these limitations, the surface of the demolded HFMN array was treated using LSPS, a mask‐free, high‐resolution digital patterning technique (Figure 1b,iii). A focused CO_2_ laser provides highly localized photothermal energy and an associated electric field, which destabilizes the core‐shell structure of the PEDOT: PSS particles. This targeted energy input induces a phase separation, forcing the insulating, hydrophilic PSS chains to segregate away from the conductive, hydrophobic PEDOT chains.^[^ ^30^, ^34^ ^]^ The PEDOT chains then reorganize and aggregate into highly interconnected, percolating networks (see Figure 1b,iii inset). This microstructural rearrangement elegantly solves two problems simultaneously: the continuous PEDOT network creates an efficient pathway for charge transport, dramatically increasing electrical conductivity, while the resulting hydrophobic surface renders the electrode layer stable in aqueous biological fluids—a critical requirement for long‐term in vivo sensing. This approach allows for higher conductivity in hydrogel‐based MNs compared to previous studies (Table S1, Supporting Information).

#### 2.1.3 Fabrication of the Dressing System

The final stage of fabrication involves processing the functionalized HFMN arrays and integrating them into the wearable dressing platform. The LSPS‐treated HFMN sheets were precisely cut into circular electrodes, a design choice that enables the creation of replaceable sensor modules for the final device (Figure 1c,i). This modularity is a key feature for enhancing clinical utility, as it allows for the convenient replacement of individual sensors that may fail due to biofouling or enzyme deactivation over time, without needing to discard the entire dressing. These individual HFMN sensor modules were then mounted onto a flexible, custom‐designed polar array substrate featuring pre‐patterned electrical contacts (Figure 1c,ii). The complete sensor assembly was then affixed to a transparent, gas‐permeable medical‐grade adhesive tape (Figure 1c,iii). The final dressing permits direct visual inspection of the underlying wound without removing the device. Moreover, the measurement setup (Figure 1d) and a recorded Video S1 (Supporting Information) showing the normal movement of animals after attaching the HFMNs further confirm its feasibility for clinical application.

### 2.2 Morphological and Physical Characterizations

**Figure 2:**
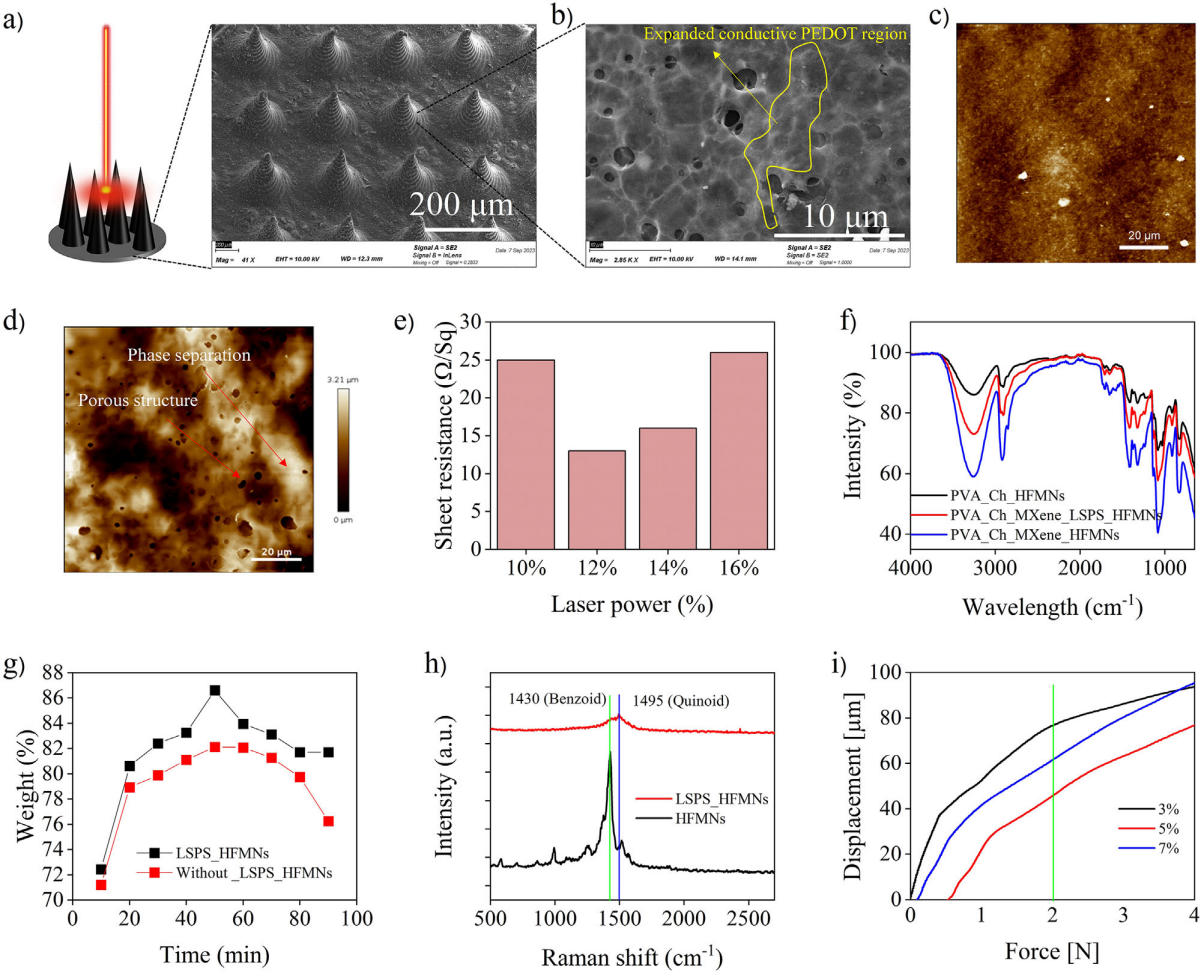
Morphological, chemical, and mechanical characterization of the HFMNs. a) SEM image of the final HFMN array after LSPS treatment, showing uniform, conically‐shaped needles with a height of ∼800 µm and a sharp tip of ∼10 µm, confirming the structural integrity of the micro‐features post‐fabrication. b) High‐magnification SEM image revealing the expanded, porous network of the PEDOT‐rich region created by the LSPS process, which provides an efficient pathway for charge transport. c,d) AFM phase images of the hydrogel surface before (c) and after (d) LSPS. The treatment transforms the surface from one dominated by insulating PSS‐rich domains (dark color) to one with highly interconnected, conductive PEDOT‐rich domains (bright color), visually confirming the phase separation. e) Optimization of laser power for the LSPS process, demonstrating that a power of 12% achieves the highest electrical conductivity (sheet resistance of 13 Ω/Sq). f) FTIR spectra confirming the chemical composition and the formation of supramolecular hydrogen‐bonding networks between the PVA, Ch, and MXene components. g) Swelling ratio analysis, indicating that the LSPS process enhances both the swelling rate and the aqueous stability of the hydrogel. Data presented as mean ± SD (*n*=3). h) Raman spectroscopy analysis showing the conformational shift in PEDOT from a less conductive benzoid structure to a highly conductive quinoid structure after LSPS, which explains the significant increase in conductivity. i) Mechanical compression testing, demonstrating that the HFMNs possess sufficient rigidity to penetrate the epidermis without failure, with the 5% MXene formulation showing optimal mechanical strength.

After the LSPS technique, HFMNs exhibited a conical structure with a length of ≈800 µm, a pointed tip measuring ≈10 µm, and an inter‐needle spacing of 700 µm, as observed by scanning electron microscopy (SEM) (**Figures** **2**a, S3, Supporting Information). This indicates that the coated laser‐scribed MNs possess uniform microstructural features after selective phase‐separation. The phase separation characteristics of the laser‐scribed hydrogel were examined in relation to the GO solution fraction in PEDOT: PSS. 2 mg mL^−1^ GO solutions (1–10 volume%) in water were sonicated with a composite PEDOT: PSS solution and dried. As we increased the GO volume %, the optimal LP decreased, indicating that a greater GO volume fraction improved laser absorption and increased thermal and electric field effects (Figure S4, Supporting Information). The relative concentration of PEDOT: PSS components, however, was inadequate to create a stable conducting network between the PEDOT‐rich regions when an excessive quantity of GO was included. The robust expanded network connecting the PEDOT‐rich region (Figure 2b) may facilitate a more efficient pathway for fast charge transfer. Atomic force microscopy (AFM) phase image analysis was used to verify the laser's selective phase separation. Prior to laser scribing, the PSS‐rich region (dark color) obscured the PEDOT‐rich domain, which was previously small and bright in color (Figure 2c). After the LSPS, the PEDOT‐rich domain significantly increased and interconnected (Figure 2d). When the laser was working properly, increasing the LP made PEDOT: PSS more electrically conductive and stable in water. But when the LP was too high, PEDOT: PSS turned into carbon, and its conductivity started to drop (Figure 2e). High‐speed laser scribing (300 s) may obtain the maximum electrical conductivity (sheet resistance of 13 Ω/Sq) for LP 12% when combined with the impact of GO.

The chemical bonding of the hydrogel is confirmed by Fourier transform infrared spectroscopy (FTIR), as illustrated in Figure 2f. The peaks at 3417, 2927, 1616, 1413, and 1029 cm^−1^ in the FTIR spectra of the PVA/Ch, PVA/Ch/MXene, and LSPS‐based PVA/Ch/MXene HFMNs correspond to the stretching vibrations of O─H, C─H, COO‐, C═O, and C─O─C, respectively.^[^ ^25^, ^35^ ^]^ It provides evidence that supramolecular H bonding networks are formed between PVA, Ch, and Ti_3_C_2_T_x_. Comparatively, the maximal intensity of HFMNs appears to have decreased marginally following LSPS as a result of an increase of hydrophobic PEDOT‐rich regions.

In order to evaluate the impact of LSPS on the swelling capacity of HFMNs, their mass changes over time were observed in SIWF. The swelling ability of HFMNs is determined by their porous morphology. The findings depicted in Figure 2g indicate that the LSPS significantly impacted the gel's water content and enhanced its swelling rate as a consequence of its porous structure. Notably, the LSPS method enhances the aqueous stability of the hydrogel through the conversion of hydrophobic PEDOT‐rich regions.

Additionally, Raman spectroscopy was employed to examine the molecular configuration of the PEDOT‐rich region (Figure 2h). The PEDOT crystallite possesses a benzoid structure (1430 cm^−1^) characterized by its relatively low electrical conductivity, as well as a quinoid structure (1495 cm^−1^) that is associated with its high electrical conductivity.^[^ ^36^, ^37^ ^]^ This conformation shift in the PEDOT morphology from a helical structure to a linear structure, also known as the secondary doping effect, was suggested by the observed decrease in benzoid and rise in quinoid structure.^[^ ^37^ ^]^

Subsequently, mechanical integrity testing was conducted in order to ascertain whether various compositions of HMN possess sufficient compressive strength to penetrate the epidermis effectively. A minimum force of 0.045 N/Needle is necessary in order to achieve epidermal piercing.^[^ ^38^ ^]^ When tested under mechanical compression, none of the MNs exhibited any indications of plastic distortion or failure, as evidenced by the force‐displacement and stress‐strain relationship that was nearly linear (Figure 2i; Figure S5, Supporting Information). An increase in MXene concentration (from 3% to 7%) resulted in a displacement of 76 µm for 3% HFMNs at 2 N. However, for 5% MXene, the recorded displacement decreased to 45 µm, indicating an enhancement in mechanical rigidity. Nonetheless, an excess of MXene 7% induced an increase in displacement of 61 µm, which might be attributed to agglomerated 3D hydrogel formation. When compared to other hydrogel MNs, our results indicated exceptional mechanical stability.^[^ ^26^, ^38^, ^39^ ^]^

### 2.3 Characterization of the HFMNs Sensor Array for Multiplexed CWO‐Biomarkers Analysis

We employed circular replaceable array sensors as an efficient method to extend the operational duration of the implantable systems, accounting for the inevitable deactivation of enzymes and mechanical friction. A polar‐type sensor array of seven electrodes was engineered utilizing laser‐scribed graphene‐based flexible electrodes with their connections passivated by a waterproof layer of plasticizer. All active circular laser‐cut HFMN‐based sensors for Glu, UA, pH, Na⁺, Cl^−^, K⁺, and T, along with the counter electrode (CE) and reference electrode (RE), are arranged in separate, replaceable modules. Hence, a replacement array may be quickly installed in the sensor platform in case an electrode is used and not working. Additionally, in a manufacturing setting, it will be simpler to change the production line from one analyte to another.

Following the brush‐painting^[^ ^16^ ^]^ of a thin layer of conductive material as the liquid adhesive onto the electrode pad, an array of functionalized HFMNs is applied for further characterizations. All electrochemical and thermal characterizations were conducted in a high‐fidelity SIWF – a complex solution of physiological salts (NaCl, KCl, CaCl_2_, etc.) and 3.3% bovine serum albumin – to emulate wound‐site ISF.^[^ ^5^, ^10^ ^]^ This wound‐like test medium contains broad analyte ranges (e.g., Glu, UA, electrolytes, and pH) similar to in vivo conditions, posing a challenge to sensor linearity. To meet this challenge, we adopted Prussian Blue (PB)‐mediated electron transfer in the enzymatic sensors and optimized diffusion‐limiting coatings.

**Figure 3:**
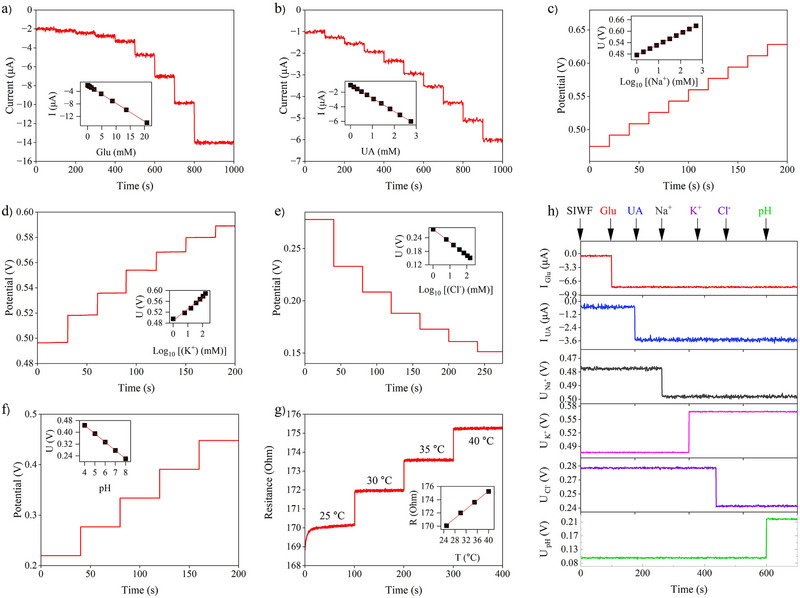
Electrochemical and thermal characterization of the multiplexed HFMN sensor array in SIWF. a,b) Chronoamperometric response curves for the enzymatic sensors upon successive additions of Glu and UA, respectively, with corresponding calibration plots shown in the insets. c–e) Potentiometric response curves for the ion‐selective electrodes to varying concentrations of Na⁺, K⁺, and Cl^−^, with respective calibration plots in the insets. f) Open‐circuit potential response of the polyaniline‐based pH sensor across the physiological pH range. g) Resistive response of the integrated platinum‐based temperature sensor to changes across a physiologically relevant temperature range, with the corresponding calibration plot shown in the inset. h) Selectivity investigation of the seven‐sensor array, illustrating the response of each sensor to the sequential addition of all target analytes to demonstrate specificity and minimal cross‐interference. All data points for calibration curves represent the mean of *n* = 3 measurements.

The Glu and UA sensors are designed as first‐generation amperometric biosensors, operating on a two‐step enzymatic principle.^[^ ^40^ ^]^ First, an oxidase enzyme—glucose oxidase (GOx) for glucose or uricase (UOx) for uric acid—catalyzes the oxidation of its specific substrate. This enzymatic reaction produces hydrogen peroxide (H_2_O_2_) as a byproduct. Second, this H_2_O_2_ is detected electrochemically. To achieve this with high sensitivity and selectivity, a layer of PB is electrodeposited onto the working electrode to act as an “artificial peroxidase” or redox mediator. PB efficiently catalyzes the reduction of H_2_O_2_ at a low applied potential of ≈0.0 V vs. Ag/AgCl.^[^ ^16^ ^]^ Operating at this low potential is a critical design feature, as it minimizes electrochemical interference from other electroactive species commonly found in biological fluids, such as ascorbic acid, ensuring high selectivity for the target analyte. The resulting reduction current is directly proportional to the H_2_O_2_ concentration and, therefore, to the concentration of glucose or uric acid in the sample. The entire assembly is covered by a porous polyurethane (PU) diffusion membrane, which helps control the flux of the analyte to the enzyme layer, thereby extending the linear response range and enhancing stability in the complex wound fluid matrix. The chronoamperometric response of the Glu sensor to successive additions of Glu into SIWF is presented in **Figure** **3**a. The sensor exhibited a robust and linear response, with the corresponding linear calibration curve shown in the inset, yielding a normalized sensitivity of −18.39 µAmM^−1^cm^−2^. Similarly, the UA sensor demonstrated excellent performance, as shown in the chronoamperometric trace in Figure 3b, with its linear calibration plot displayed in the inset. This sensor yielded a normalized sensitivity of −57.87 µAmM^−1^cm^−2^. The significantly higher sensitivity for UA is advantageous for accurately quantifying its lower physiological concentrations in the wound environment.

The potentiometric sensor suite is designed to measure ion concentrations and pH based on the principles of ion‐selective electrodes (ISEs).^[^ ^41^ ^]^ Each sensor generates a potential difference at the interface between an ion‐selective membrane (ISM) and the sample solution, which is measured against a stable Ag/AgCl reference electrode. This potential is logarithmically related to the activity of the target ion, following the Nernst equation.

1. Na⁺ and K⁺: These sensors rely on specific ionophores embedded within a polymeric membrane. The Na⁺ sensor, which utilizes Sodium Ionophore X, displayed a stable and linear potentiometric response to varying analyte concentrations (Figure 3c). As derived from the linear calibration curve in the inset, the sensor exhibited a near‐Nernstian sensitivity of +56.8 mV/decade. The K⁺ sensor, based on the selective ionophore valinomycin, demonstrated a consistent sensitivity of +43.17 mV/decade (Figure 3d).
2. Cl^−^ Sensor: The Cl^−^ sensor, operating on the principle of an Ag/AgCl second‐kind electrode, also showed a highly selective, near‐Nernstian response of −57.7 mV/decade (Figure 3e), with its linearity confirmed by the inset plot.
3. pH Sensor: The pH sensor (Figure 3f) employed an electropolymerized polyaniline (PANI) film, a conducting polymer whose potential is directly dependent on H⁺ concentration. In the McIlvaine buffer and SIWF, it showed a near‐Nernstian potential response of ≈−57.0 mV/decade unit between pH 4 and 8, with excellent linearity (R^2^ ≈ 0.99). A miniaturized Ag/AgCl reference electrode was included to provide a stable reference in the buffering SIWF environment.

To monitor local wound temperature, an integrated resistive T sensor is included. This sensor operates on the principle of a resistive temperature detector (RTD), where the electrical resistance of a metallic element changes linearly with temperature.^[^ ^42^ ^]^ This allows for precise thermal measurements, with the sensor exhibiting a sensitivity, or temperature coefficient of resistance (TCR), of 0.343 %°C^−1^. The stepwise response to temperature changes and the corresponding linear calibration plot are shown in Figure 3g. This integrated thermal sensing is crucial not only for its diagnostic value but also for enabling real‐time computational correction of the entire electrochemical sensor array, thereby compensating for the thermal dependence of enzymatic and potentiometric measurements and ensuring high analytical accuracy for the overall system.

The selectivity of the entire sensor array is a critical parameter for reliable multiplexed analysis in a complex medium. Figure 3h illustrates the results of the selectivity investigation, where the response of each sensor was monitored during the sequential addition of potentially interfering species to the SIWF. The data clearly demonstrates that each sensor responds exclusively to its target analyte. The amperometric Glu and UA sensors showed a distinct signal change only upon the addition of glucose and uric acid, respectively, with no discernible response to the other ions or changes in pH. Likewise, the potentiometric sensors for Na^+^, K^+^, Cl^−^, and pH each exhibited a significant potential shift only when their specific target analyte was introduced. This negligible cross‐interference across the entire sensor suite confirms the high selectivity of the individual sensing elements and validates the platform's suitability for accurate, simultaneous monitoring of multiple biomarkers in a clinically representative environment.^[^ ^40^, ^43^ ^]^

All functionalized HFMN sensors demonstrated high stability, retaining consistent calibration slopes for at least five days of continuous exposure to SIWF. While the sensors remained functional for up to seven days (Figures S6–S12, Supporting Information), continued swelling of the hydrogel after the fifth day began to gradually alter diffusion profiles and reduce sensitivity. In summary, the HFMN sensor array exhibited quantitative, linear responses over the physiologically relevant SIWF ranges for Glu, UA, pH, Na^+^, Cl^–^, K^+^, and temperature, with sensitivities and slopes consistent with Nernst theory and with published values.^[^ ^16^ ^]^ These results demonstrate that the integrated PB‐mediated enzymatic sensors and ion‐selective electrodes can reliably monitor chronic wound biomarkers in situ.

Additionally, to establish a robust performance baseline for the comparative in vivo analysis, the conventional surface‐electrode‐based sensor patch used in subsequent animal studies was also characterized under identical simulated wound conditions. This validation step confirmed that the benchmark device was fully functional and capable of quantitative analysis across the relevant physiological ranges. The results (Figure S13, Supporting Information) confirm that all target analytes—Glu, UA, Na⁺, K⁺, Cl^−^, pH, and T—were successfully detected with comparable sensitivity and linearity.

### 2.4 Antibacterial Activity and Therapeutic Capabilities

An ideal wound dressing must possess a dual capability: to actively support the host's cellular repair mechanisms while simultaneously preventing or eliminating bacterial infections that can impede healing. A comprehensive in vitro investigation was therefore conducted into the therapeutic and antibacterial efficacy of the HFMNs, focusing on their ability to modulate fibroblast behavior and inhibit pathogenic bacteria.

**Figure 4:**
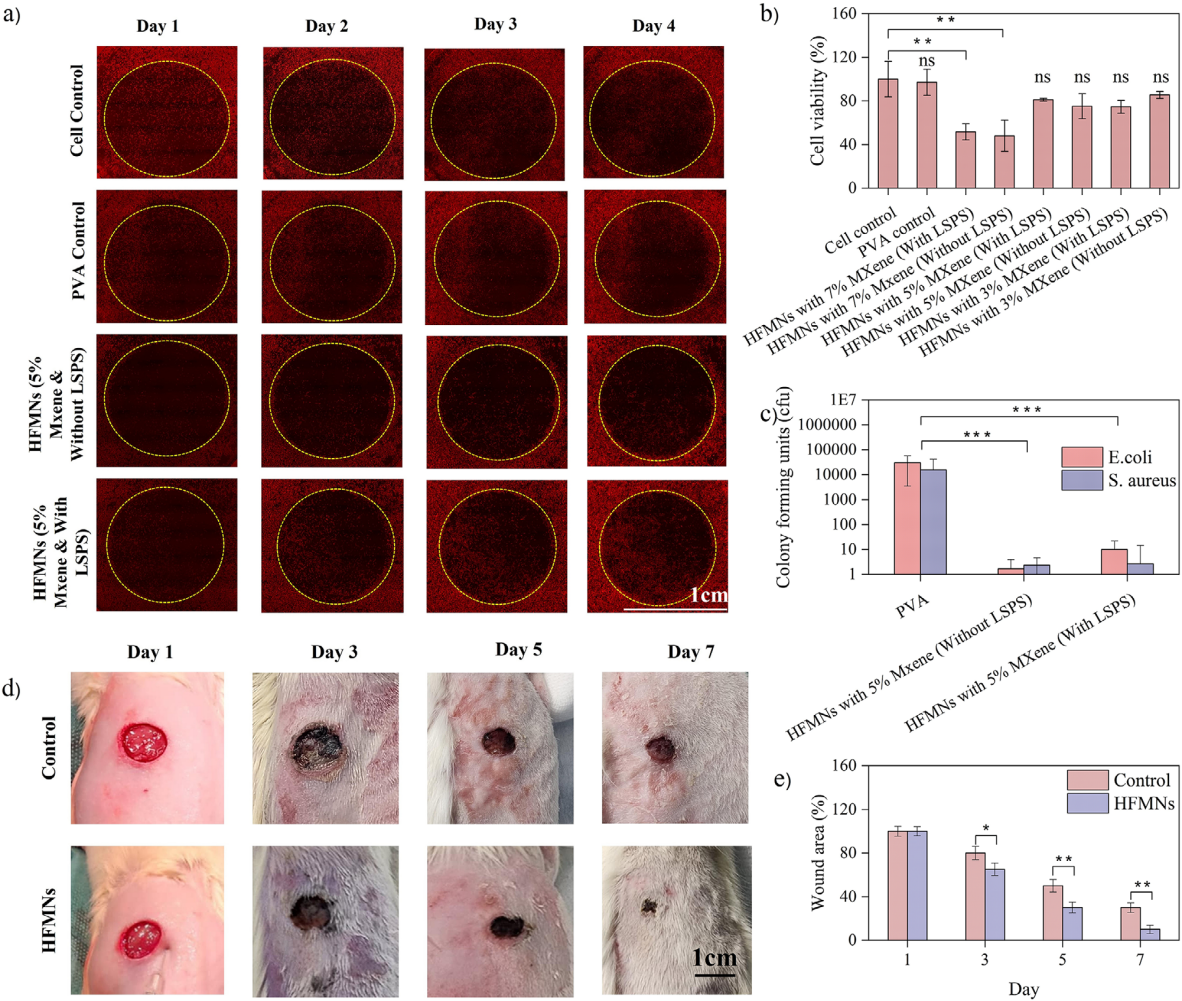
In vitro and in vivo evaluation of the therapeutic capabilities of the HFMN dressing. a) Fluorescence microscopy images from an in vitro wound healing assay, illustrating fibroblast migration and wound gap closure over four days. b) Quantitative assessment of fibroblast viability via a WST‐8 assay. Data are presented as mean ±SD (*n* = 3). ** *p* < 0.01 vs. Cell Control. Statistical analysis was performed by one‐way ANOVA with Tukey's post‐hoc test. c) Graphical analysis of antimicrobial activity against *E. coli* and *S. aureus*. Data are presented as mean ±SD (*n* = 3). ***p < 0.001 vs. PVA Control. Statistical analysis was performed by one‐way ANOVA with Tukey's post‐hoc test. d) Photographs documenting the visual progression of wound healing in an animal model over seven days. e) Graphical analysis quantifying the rate of wound area reduction over time. Data are presented as mean ±SD (*n* = 4 rats per group). *p < 0.05, **p < 0.01, ***p < 0.001 vs. Control group at the corresponding time point. Statistical analysis between groups was performed by two‐way ANOVA with Tukey's post‐hoc test.

The pro‐regenerative potential of the hydrogel formulations was evaluated using an in vitro wound healing assay, which tracks the migration and proliferation of fibroblasts to close a simulated wound gap created by gelatin dissolution. The qualitative results are presented as a series of fluorescence microscopy images in **Figure** **4**a, where the initial wound area is demarcated by yellow‐dotted circles. Over the course of four days, a consistent and expected progression of wound closure was observed in the cell control and PVA control groups, with fibroblasts steadily migrating and proliferating to form a confluent monolayer. In comparison, only moderate healing was observed for the HFMNs containing 5% MXene without LSPS, with the wound gap being less completely closed than the controls by Day 4. Most notably, the group treated with the 5% MXene HFMNs fabricated via LSPS exhibited the most effective and accelerated healing. This finding is consistent with reports that MXene‐containing dressings promote rapid re‐epithelialization and robust tissue regeneration.^[^ ^44^, ^45^ ^]^

Cell viability measured by WST‐8 (Figure 4b) showed that moderate MXene loading (5 wt.%) with LSPS yielded high fibroblast viability (≈80% after 7 days), while very high loading (7%) reduced viability (≈50%) due to strong antibacterial and potential cytotoxic effects. Interestingly, the LSPS process offers a critical advantage in this trade‐off: by partially reducing the surface functional groups of MXene during LSPS, it slightly decreases the sharp antibacterial potency but significantly improves hydrogel stability and biocompatibility within the wound environment. This balance allows the LSPS‐processed HFMNs to maintain sufficient antimicrobial action while supporting cellular proliferation and tissue repair. Recent studies have similarly noted that modifying MXene surface chemistry—either by thermal treatment or laser processing—reduces reactive oxygen species generation and sharp‐edge effects, thereby enhancing cytocompatibility without fully compromising antimicrobial activity.^[^ ^45^, ^46^, ^47^ ^]^ Overall, the 5% MXene with LSPS formulation demonstrates an optimal compromise between antibacterial efficacy and cytocompatibility, ensuring safe and effective wound healing.

Bacterial counts (Figure 4c) demonstrate that MXene‐containing hydrogels exhibit strong antimicrobial action, with the formulation without LSPS showing slightly higher bactericidal activity compared to the LSPS‐treated variant. Both Gram‐negative *E. coli* and Gram‐positive *S. aureus* were substantially reduced by MXene‐based hydrogels relative to PVA alone, achieving >95% mortality in the non‐LSPS group. This is consistent with MXene's broad‐spectrum antibacterial mechanisms: its sharp nanosheets physically disrupt bacterial membranes, while surface terminations (─OH, ─F, ─O) facilitate ROS generation and photothermal effects under NIR irradiation.^[^ ^48^, ^49^ ^]^ Literature supports these observations, for instance, MXene–PEDOT scaffolds eradicated *E. coli* and *S. aureus* (even MRSA) in vitro while promoting angiogenesis,^[^ ^50^ ^]^ and anisotropic MXene@PVA hydrogels prevented wound infections in rat.^[^ ^25^ ^]^ In our system, while the LSPS process slightly reduces MXene's direct antimicrobial activity by modifying surface functionalities, it enhances the hydrogel's structural stability and biocompatibility. This trade‐off ensures that the LSPS‐treated HFMNs maintain sufficient antimicrobial action while supporting tissue repair, as highlighted in Figure 4b.

In the rat wound model (Figure 4d), gross healing was observed to progress significantly faster with HFMN treatment. By Day 1, both the control and HFMN‐treated groups exhibited equivalent fresh wounds of similar size and appearance. By Day 3, the control wounds developed thick scabs and noticeable redness, indicative of ongoing inflammation, whereas the HFMN‐treated wounds displayed thinner scabs and early signs of re‐epithelialization accompanied by reduced inflammation. By Day 5, the treated group exhibited a substantially larger area of healed tissue and enhanced skin integration, while the control group still presented prominent scabs and incomplete closure. By Day 7, the wounds in the HFMN group were nearly fully closed, displaying a smooth and uniform surface, in contrast to the darker, partially open wounds persisting in the control group. These observations align with previous reports where MXene‐based MN patches were found to reduce inflammatory markers and promote angiogenesis, collagen deposition, and re‐epithelialization in infected wound models.^[^ ^51^ ^]^ The accelerated closure and reduced inflammation observed with LSPS‐HFMNs suggest that the conductive and antibacterial properties of the dressing synergistically modulate the wound microenvironment to facilitate regeneration, consistent with other MXene‐doped wound therapies.^[^ ^52^ ^]^

The quantitative analysis of wound area reduction over time (Figure 4e) further reinforces the advantage of LSPS‐HFMNs. Wounds treated with LSPS‐HFMNs exhibited significantly faster shrinkage, with wound areas dramatically smaller than those of the controls by Day 7. Statistical analysis confirmed a significantly greater percentage of wound closure at each timepoint. This pattern corresponds with findings from other quantitative studies of MXene‐based dressings, where enhanced closure was attributed to multifunctional properties. In this case, the improved healing is likely the result of LSPS‐HFMNs providing synergistic effects—combining electrical conductivity, moisture retention, and potent antimicrobial activity—which collectively accelerate granulation tissue formation, wound contraction, and epithelial coverage. Both visual and numerical data indicate that LSPS‐HFMNs act as multifunctional wound dressings capable of orchestrating antibacterial defense, promoting tissue regeneration, and enhancing extracellular matrix formation for more complete and rapid wound healing.

### 2.5 In Vivo Monitoring and Correlation: Validating Dermal Wound Fluid as a High‐Fidelity Biofluid for Wound Diagnostics

**Figure 5:**
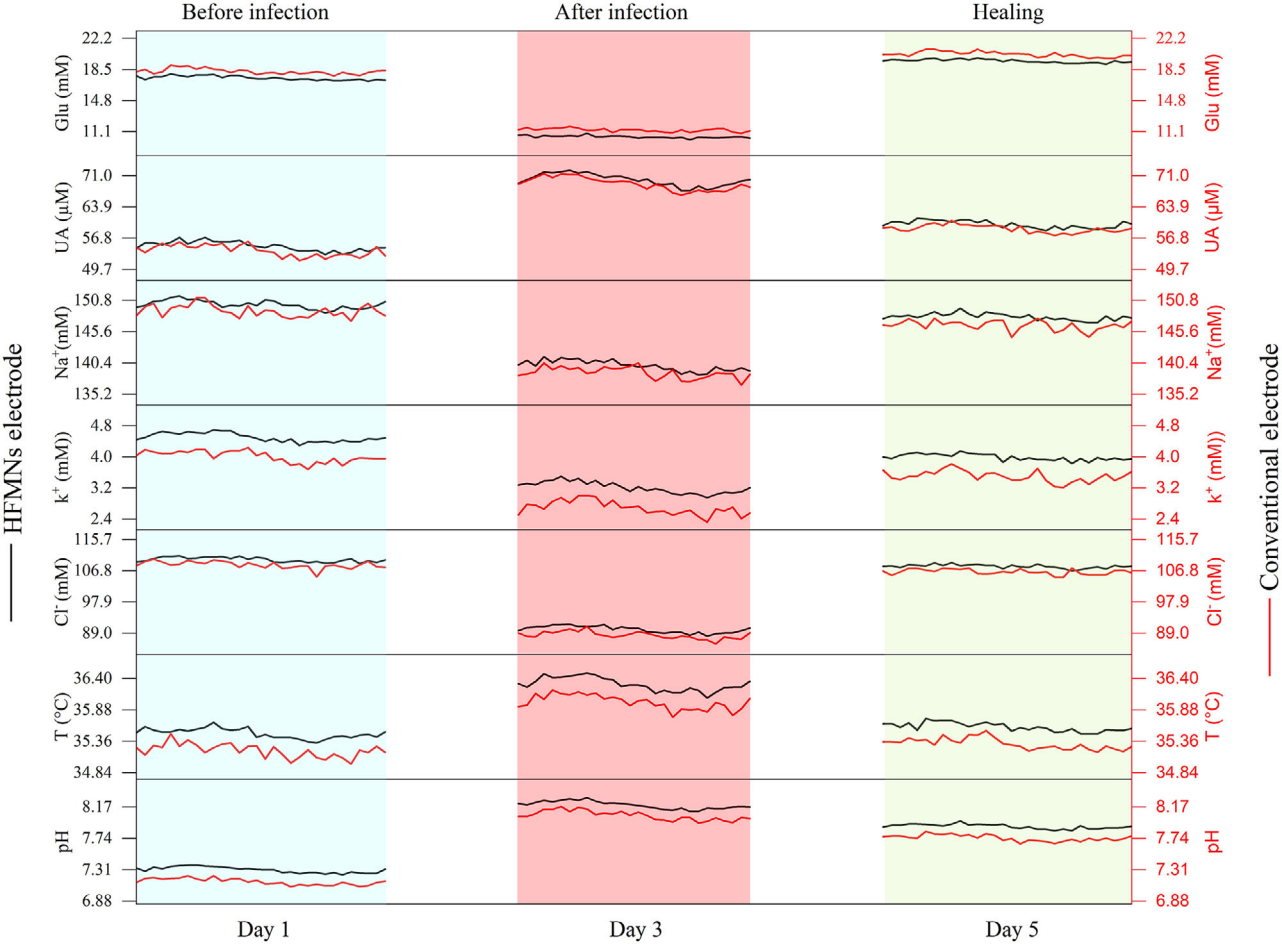
Comparative in vivo monitoring of wound biomarkers, validating dermal wound interstitial fluid as a high‐fidelity diagnostic source. This figure presents a direct comparison of real‐time data collected from the HFMN dressing (sampling dermal wound ISF, black lines, *n* = 5) and a conventional surface sensor patch (sampling wound exudate, red lines, *n* = 5) in a rat model. Measurements were taken at three critical stages: pre‐infection (Day 1), peak infection (Day 3), and healing (Day 5). The plots display the dynamic changes of seven key biomarkers: Glu, UA, Na⁺, K⁺, Cl^−^, pH, and T. Data points represent the mean from *n* = 8 rats, with error bars indicating SD.

In vivo testing of the HFMN dressing (**Figure** **5**) was performed in a wound model at three time points – pre‐infection (baseline, Day 1), post‐infection (peak inflammation, Day 3), and healing (Day 5). In this experiment, the HFMN sensor array continuously sampled dermal wound fluid through MNs, while a conventional adhesive patch with surface electrodes sampled wound exudate on the skin surface. By accessing dermal wound fluid, the HFMNs captured deeper tissue biochemistry, whereas the topical sensors reflected only surface exudate. Figure 5 directly compares the two approaches: the HFMN data generally showed the same trends as exudate measurements but with more physiologically informative changes, consistent with higher dermis‐level specificity.

For Glu, both systems started at comparable baseline levels (HFMNs: 17.93 mm, exudate: 17.95 mm). By Day 3, Glu levels had dropped significantly to 10.65 mm (HFMNs) and 11.09 mm (exudate), reflecting bacterial consumption of Glu during infection.^[^ ^53^ ^]^ By Day 5, Glu rebounded above baseline in both systems (HFMNs: 19.20 mm, exudate: 20.09 mm), consistent with restored tissue perfusion and reduced bacterial load.^[^ ^53^ ^]^ Notably, conventional electrodes recorded a slightly earlier and larger Glu rebound than the HFMNs sensor.

UA levels showed a distinct increase post‐infection (Day 3) due to xanthine oxidase activity and inflammatory response.^[^ ^54^, ^55^ ^]^ UA rose from baseline values (HFMNs: 53.97  µm, exudate: 55.02  µm) to 69.15 µm (HFMNs) and 70.05 µm (exudate) on Day 3, maintaining this level through Day 5 (HFMNs: 58.96  µm, exudate: 59.97 µm).

Electrolyte dynamics were also pronounced. Na⁺ decreased from Day 1 (HFMNs: 149.67 mm, exudate: 148.26 mm) to Day 3 (HFMNs: 140.07 mm, exudate: 138.32 mm), then rose again on Day 5 (HFMNs: 147.75 mm, exudate: 146.71 mm) as homeostasis was restored. K⁺ levels similarly dropped during infection (Day 3: HFMNs: 3.27 mm, exudate: 2.51 mm) from baseline (HFMNs: 4.44 mm, exudate: 4.04 mm) and partially recovered by Day 5 (HFMNs: 4.00 mm, exudate: 3.66 mm). Cl^−^ followed a comparable trend with Day 1 values (HFMNs: 109.33 mm, exudate: 108.31 mm) decreasing to Day 3 (HFMNs: 89.72 mm, exudate: 89.08 mm) and rebounding by Day 5 (HFMNs: 108.08 mm, exudate: 106.77 mm). These changes reflect cellular lysis and tissue remodeling during infection and healing.^[^ ^8^ ^]^

For pH, an alkaline shift was observed during infection, rising from baseline (HFMNs: pH 7.33, exudate: pH 7.14) to peak values on Day 3 (HFMNs: pH 8.21, exudate: pH 8.04). By Day 5, pH normalized to near pre‐infection levels (HFMNs: pH 7.89, exudate: pH 7.76), tracking the resolution of infection.^[^ ^56^ ^]^

Temperature measurements confirmed local inflammatory response. Baseline wound temperatures (hydrogel electrode: 35.51 °C, conventional electrode: 35.25 °C) rose on Day 3 (hydrogel electrode: 36.31 °C, conventional electrode: 35.93 °C) and returned close to baseline by Day 5 (hydrogel electrode: 35.65 °C, conventional electrode: 35.35 °C), consistent with inflammation resolution.^[^ ^57^ ^]^

Overall, while both sensor types exhibited similar qualitative trends, the HFMN system demonstrated noticeably sharper responses for several biomarkers, providing earlier and more distinct insights into wound biochemistry. Dermal wound fluid sampling enabled real‐time detection of pathophysiological changes at their source, offering superior resolution over surface exudate. These results validate the HFMN dressing as a reliable platform for precision wound diagnostics and personalized care.

## 3 Conclusion

We successfully developed and validated a theranostic transdermal wound dressing system based on highly conductive HFMNs. By using LSPS technique directly on a soft hydrogel, we have overcome the long‐standing challenge of creating robust, water‐stable, and highly conductive (384 S/m) electrodes on a biocompatible platform. This system uniquely integrates advanced diagnostics with active therapy. The MXene‐integrated hydrogel matrix was shown to be cytocompatible, possess potent antibacterial properties against both *E. coli* and *S. aureus*, and significantly accelerate wound closure in an in vivo rat model. The multiplexed sensor array reliably monitored key wound biomarkers—Glu, UA, Na⁺, K⁺, Cl^−^, pH, and T—with high selectivity and stability in a complex, protein‐rich simulated wound fluid. Most critically, our comparative in vivo study demonstrated that by accessing dermal wound interstitial fluid, the HFMN dressing provides a more immediate and higher‐fidelity biochemical profile of the wound's status compared to conventional sensors that sample surface exudate. This work validates dermal ISF as a superior source for real‐time wound diagnostics. While this platform establishes a new paradigm for smart wound care, this success paves the way for future translational development. The next phase of optimization will naturally focus on extending the in situ stability of the multiplexed array beyond the demonstrated 5–7 days, a key step toward long‐term chronic wound management. From a manufacturing perspective, the innovative multi‐stage, hybrid fabrication—which proved highly effective at the laboratory scale—now presents clear opportunities for process engineering to achieve high‐throughput scalability and reproducibility. Finally, future work can explore pathways to optimize the economic feasibility of the replaceable sensor modules, for instance, by streamlining production or assessing alternative advanced materials, which will be crucial for ensuring broad clinical and commercial adoption. This fully integrated platform represents a significant step forward, establishing a new paradigm for smart wound care that directly links deep‐tissue biochemistry with therapeutic action. We foresee this technology enabling a shift from reactive treatment to proactive, personalized management of chronic wounds.

## 4 Experimental Section

### Materials and Reagents

MAX phase (Ti_3_AlC_2_) (≥40 µm) powder, potassium tetrachloroplatinate, cyclohexanone, tetrahydrofuran, bis(2‐ethylhexyl)Sebacate (DOS), N‐Hydroxy succinimide (NHS), N‐(3‐Dimethylaminopropyl)‐ethylcarbodimide hydrochloride (EDC), polyurethane, graphene Oxide, poly(3,4‐ethylenedioxythiophene)‐poly(styrenesulfonate) (PEDOT: PSS), aniline [C_6_H_7_N], chitosan, D‐glucose, sucrose, uric acid, poly vinyl chloride (PVC), poly vinyl alcohol (PVA), glucose oxidase from *Aspergillus* *n* *iger*, uricase from *Candida Sp*., sodium‐(triflutomethyl)Phenylborate[Na‐TFPB], bovine serum albumin (BSA), selectophore grade sodium ionophore X, sodium tetrakis[3,5‐bis(trifluoromethyl)phenyl] borate (Na‐TFPB), valinomycin (potassium ionophore), sodium tetraphenylborate (NaTPB), dimethyl sulfoxide (DMSO), potassium ferricyanide (K_3_Fe(CN)_6_), HCl, H_2_SO_4_, glutaraldehyde solution (20−25%), poly(vinyl butyral), sodium chloride (NaCl), potassium chloride (KCl), calcium chloride (CaCl_2_), sodium bicarbonate (NaHCO_3_), sodium phosphate (NaH_2_PO_4_), magnesium sulfate (MgSO_4_), sodium gluconate (NaC_6_H_11_O_7_), lactate, buffer solution (pH = 4, 7, and 10), PBS (pH = 7.2), and Sylgard 184 elastomer kit (PDMS and curing agents) were bought from Sigma‐Aldrich (Germany). All solutions were prepared using deionized water produced from a Millipore water purification system, unless otherwise noted.

### Fabrication of the MN Array

AutoCAD was utilized in the development of the computer‐aided design (CAD) file. The CAD was comprised of a circular base measuring 60 × 40 mm film (5 mm in thickness), which featured 49 × 43 conically‐shaped needles with an interneedle spacing of 700 µm and a base diameter of 300 µm and a height of 800 µm, respectively. Following its exportation as a standard tesselation language (.STL) file, the document was imported into the Preform 3D printing application developed by FormLabs, USA. Prior to generating supports with a reduced touchpoint size and configuring the high‐temperature resin to print at a high resolution, the component was initially inclined at an angle of 45°. After the printing process, the resulting prints underwent a 60‐min washing in IPA with FormWash, followed by a 150‐min treatment at 70 °C under 405 nm UV with FormCure. A scalpel was employed to remove the supports by means of a gentle break at the interface where the supports met the base of the MN array.

### Fabrication of HFMNs

HFMNs involve a total four vital phases: i) preparing the homogenous hydrogel solution, ii) pouring it into a PDMS female mold, iii) cross‐linking by freezing followed by thawing, furthermore, iv) drying and demolding the MN patch. In the first phase, 15% (w/w) PVA solution was prepared with the addition of 3%, 5%, and 7% MXene solution, heated at 90 °C for 3 h. For the synthesis of MXene, the modified minimally intense layer delamination MILD synthesis method, as described in the prior publication, was utilized.^[^ ^33^ ^]^ Separately, 1% (w/w) Ch solution was prepared in 0.1 m acetic acid at room temperature (RT). Subsequently, PVA/Ch solution ratio (4:1) was mixed, and stirred overnight at RT. The PVA/Ch solution was subsequently injected into a PDMS female mold applying a vacuum to fill the MN pores in the second phase. Furthermore, the hydrogel forming MNs (HFMNs) were frozen at −20 °C for 3 h and thawed at room temperature, then dried in oven at 50 °C for 6–8 h. Afterward, the MNs patch was removed carefully from PDMS mold.

A GO solution (2 mg mL^−1^ GO) was sonicated with PEDOT: PSS aqueous solution at a concentration of 1–10 volume% in water. The final composite was applied to the HFMNs array after 1‐h of sonication at room temperature. The array was then allowed to dry for 24 h at RT.

The laser system comprised a 10.6 µm CO_2_ laser that was employed to scribe selective phase separation via laser processing. For a final PEDOT: PSS containing 5% GO, the optimal conditions in this investigation were 12% LP and 300 mm s^−1^ scanning speed. Following laser treatment, DI water was used to rinse the samples.

### Fabrication of the Transdermal Dressing System

The methodology for producing the electrode arrays was illustrated in Figure 1. Using AutoCAD, the distinctive polar base electrode patterns were designed. The patterned 3D porous laser‐induced graphene was subsequently patterned onto commercial PI films using the CO_2_ laser scribing technique (optimized parameters: power: 14%, speed: 250 mm s^−1^). A vacuum‐degassed PDMS solution (base: curing agent = 10:1) was subsequently poured onto the scaffold containing the pattern. The pattern was carefully picked up by the PDMS film following a successful 2‐h thermal curing process at 70 °C. Next, SEBS was employed for passivation. The patch was then transferred to a transparent and breathable medical‐grade tape (Tegaderm) and applied to the subject's wound region.

### Electrochemical Characterization

A PalmSens potentiostat was utilized to assess the linear range, sensitivity, and reproducibility of the multiplex sensors. All electrochemical characterizations were conducted in a high‐fidelity SIWF to emulate the wound‐site environment. The SIWF was prepared in accordance with the previously described methodology and consisted of a complex solution of physiological salts (NaCl, KCl, CaCl_2_, etc.) and 3.3% bovine serum albumin.^[^ ^16^, ^58^ ^]^

### Fabrication of the Replaceable Sensors

1. Enzymatic Sensors: For the Glu and UA sensors, a PB layer was electrodeposited onto the working electrodes to serve as a low‐potential redox mediator. This was achieved by cyclic voltammetry in a freshly made solution containing 2.5 mm iron (III) chloride, 2.5 mm K_3_[Fe(CN)6], 100 mm KCl, and 100 mm HCl. The potential was scanned between −0.2 and 0.6 V (vs. Ag/AgCl) at 50 mV/s for 20 cycles for both sensors. A solution containing 5.05 mg Ch (in acetic acid and DI water) was combined with 10 mg of either GOx or UOx. Following 5 min stirring, 10 µL of the enzyme‐Ch solution was dropped onto the PB‐modified HFMNs surface and dehydrated at 4 °C for 24 h. The working electrode was subsequently coated with PU (1%, 2%, and 5%) as a porous, diffusion‐limiting membrane. Following a 20‐min drying period at RT, the sensor was stored at 4 °C until use.
2. Ion‐Selective and pH Sensors: The HFMNs electrode was coated with Ag/AgCl by hand, which was then cured at 120 °C for 5 min in order to produce reference electrodes for the Glu, UA, and pH sensors. In their unaltered state, HFMNs were utilized as the counter electrode. Membranes for ion sensors were produced in accordance with an earlier report.^[^ ^59^ ^]^ The components of the Na^+^ selective membrane mixture were DOS (65.45%), PVC (33%), Na‐TFPB (0.55%), and Na ionophore X (1%). The K^+^‐selective membrane contained valinomycin (2% w/w), NaTPB (0.5%), PVC (32.7% w/w), and DOS (64.7% w/w). Ag NPs were deposited for the Cl^−^ ion membrane, and FeCl_3_ was subsequently employed to chlorinate it. The HFMNs were subsequently treated with ion‐selective membranes developed by drop‐casting 8 µL of the Na+ and K+ selective membrane mixture. The HFMNs electrode was immersed in PANI precursor (0.1 m aniline aqueous solution with 1 m HCl) in order to electropolymerize the pH sensor. Then, 50 segments of CV were conducted at a scan rate of 100 mV s^−1^.
3. Temperature Sensor: Pt NPs were deposited, and an interdigitated heater array was used to create the T sensor.  The sensor was subsequently coated with PU as a protective membrane.

### Electrical Measurement

The sheet resistance of hydrogels was measured using a four‐point probe. Using the thickness data acquired earlier, the electrical conductivity was calculated with the following equation

(1) $$\sigma =\frac{1}{Rs.t}$$

where, σ, Rs, and t are conductivity, sheet resistance, and thickness.

### Mechanical Properties and Penetration of HFMNs

The dynamic mechanical analyzer (DMA) was employed to assess the mechanical properties of the conductive HFMNs. An upper titanium plate was lowered until contact was made with the MN points, as indicated by a force reading on the apparatus screen, while the MNs were affixed to the lower titanium plate. The displacement produced when a compressive force ranging from 0 to 8 N was issued from the bottom plate at a rate of 0.20 N min^−1^ was measured. To observe the impact of compression on the MNs, images were captured of the samples subsequent to the testing process.

### In Vitro Cytotoxicity and Biocompatibility Studies

The cytocompatibility of the HFMNs was assessed using primary human dermal fibroblasts (HDFs) and normal human epidermal keratinocytes (NHEKs). For metabolic activity, 1x10^5^ cells were seeded into the wells of 24‐well plates. After 24 h of incubation, the HFMN dressings were suspended in 300 µL of D10 medium in permeable transwell inserts (Corning, Costar) above the seeded cells. The plates were incubated at 37 °C and 5% CO_2_ for 7 days. On day 7, 100 µl of WST‐8 solution (Cell Counting Kit‐8, Sigma–Aldrich) was added to each well, and plates were incubated for 1 h. The absorbance at 450 nm was measured using a microplate reader, and the results were normalized to the cell control. For viability, a commercial calcein AM/ethidium homodimer‐1 live/dead kit (Invitrogen) was used. Live cells were stained green with calcein‐AM, and dead cells were stained red with ethidium homodimer‐1. Images were captured with an Axio Observer inverted microscope (ZEISS), and cell viability was calculated as the percentage ratio of live cells to the total number of cells using ImageJ software.

### In Vitro Antimicrobial Studies

Antimicrobial testing was performed in the Institute for Infection Medicine, Kiel, Germany according to previous report.^[^ ^60^ ^]^ In brief, 10 µL of an overnight culture of *E. coli* and *S. aureus* were incubated with 5 mL LB medium at 37 °C for 3 h. Subsequently, the bacteria number was estimated by measuring the optical density at 600 nm. 10 µL of a suspension of 10^5^ bacteria/ml in 0.85% NaCl and 1% LB‐medium was placed on the surface of the PVA membranes. The membranes were incubated in Petri dishes at 37 °C. After 6 h, the membranes were transferred into 1 mL 1% LB medium with 0.85% NaCl and vortexed for 30 s. Serial dilutions of the supernatant were plated on LB agar, and the resulting bacterial colonies were counted to determine the number of surviving bacteria.

### Animal Studies

All animal procedures were approved by the Ministry of Agriculture, Rural Areas, Europe and Consumer Protection, Schleswig‐Holstein, Germany (permit IX 554–70306/2024 (59‐9/24)), in accordance with EU Directive 2010/63. Experiments were performed in rats, 8–12 weeks old. Animals were anesthetized by inhalation of isoflurane (induction at 3–4%, maintenance at 1.5–2% in O_2_).^[^ ^61^ ^]^ Pre‐ and post‐operative analgesia included subcutaneous carprofen (5–10 mg kg^−1^) and oral tramadol. Tramadol was administered in the drinking water at 0.1 mg/mL (≈15–20 mg kg^−1^/day), starting immediately after surgery. Carprofen (5–20 mg kg^−1^ SC) was given once pre‐operatively and repeated as needed.

For the in vivo theranostic study, a two‐stage procedure was employed. First, a full‐thickness 13 mm diameter excisional wound was created on the dorsum of each rat under aseptic conditions. To establish a preliminary model of localized dermal infection and to test the therapeutic efficacy of the HFMNs, a bacterial challenge was administered immediately following wounding. A mixture of clinically relevant bacteria was applied to the tips of the HFMN patch.^[^ ^16^ ^]^ The patch was secured with a transparent semi‐occlusive dressing.

Patches were kept in place for the study duration (up to 7 days), and rats were monitored daily for health and patch function. Wound healing (closure, redness, swelling) and sensor signals (Glu, UA, ion concentrations, T) were recorded at defined intervals corresponding to pre‐infection (Day 1, baseline), peak infection (Day 3), and healing (Day 5). At the study's conclusion, rats were euthanized under deep isoflurane anesthesia.

### Statistical Analysis

All experiments were conducted with at least three independent replicates (*n* ≥ 3) unless otherwise stated. Raw data were initially screened to identify and remove outliers using standard deviation‐based filtering, followed by normalization to ensure comparability across experiments. Data are presented as mean ± standard deviation (SD) throughout the manuscript. For in vitro experiments, including cell viability (Figure 4b) and antimicrobial assays (Figure 4c), a sample size of *n* = 3 independent experiments were used for each condition. The in vivo wound healing study (Figure 4d,e) was conducted over 7 days with a sample size of *n* = 4 rat per group (control and HFMN‐treated). For the comparative in vivo monitoring (Figure 5), data were collected from *n* = 8 rats.

In Figure 4b, a one‐way analysis of variance (ANOVA) performed across all eight experimental groups revealed a significant effect of the treatment on fibroblast viability (*p* < 0.0001). Post‐hoc analysis using Tukey's test confirmed that high MXene loading (7%), both with and without LSPS, resulted in a statistically significant reduction in viability (≈50%) compared to the cell control *(p* < 0.01). In contrast, the “HFMNs with 5% MXene (With LSPS)” formulation (Mean = 81.04) yielded high fibroblast viability and was not statistically different from the “Cell control” (Mean = 100).

For Figure 4c, antimicrobial activity was statistically assessed using a one‐way ANOVA with Tukey's post‐hoc test. For both Gram‐negative *E. coli* (PVA control Mean = 30360) and Gram‐positive *S. aureus* (PVA control Mean = 15730), the analysis confirmed that both MXene‐based hydrogels achieved a highly significant reduction in bacterial colonies (*p* < 0.0001 vs. control). Specifically, the “5% MXene (Without LSP)” group (Mean *E. coli* = 1.67, Mean *S. aureus* = 2.33) and the “5% MXene (With LSPS)” group (Mean *E. coli* = 10, Mean *S. aureus* = 2.67) both virtually eliminated the bacterial presence.

For the quantitative analysis of wound area reduction in Figure 4e, a two‐way ANOVA was conducted (*n* = 5), demonstrating a highly significant treatment effect (*p* < 0.0001) and a significant treatment–time interaction (*p* < 0.001). This significant interaction confirms that the rate of wound closure was significantly faster in the LSPS‐HFMNs group. Post‐hoc analysis (Tukey's test) confirmed that the percentage of wound closure was statistically significant between the two groups starting from Day 3 (*p* < 0.05), with the difference becoming more pronounced at Day 5 (*p* < 0.01) and Day 7 (*p* < 0.001).

All statistical analyses were performed using OriginPro (OriginLab Corporation, Northampton, MA, USA).

### Ethical Statement

All animal procedures were approved by the Ministry of Agriculture, Rural Areas, Europe and Consumer Protection, Schleswig‐Holstein, Germany (permit IX 554–70306/2024 (59‐9/24)), in accordance with EU Directive 2010/63.

## Conflict of Interest

The authors declare no conflict of interest.

## Supporting information



Supporting Information

Supplemental Video 1

## Data Availability

The data that support the findings of this study are available in the supplementary material of this article.
