# Supplementary material for: Hydrogel Microneedle Array‐Based Transdermal Dressing System for Multiplexed Assessment and Intelligent Therapy of Chronic Wounds
Source: Small. 2025 Dec 15;22(10):e11542. doi: 10.1002/smll.202511542 (PMC12910427; doi:10.1002/smll.202511542)
Supplement: Supplementary file 1 — Supporting Information [file SMLL-22-e11542-s001.pdf]

Hydrogel Microneedle Array-Based Transdermal Dressing System for Multiplexed Assessment and Intelligent Therapy of Chronic Wounds

*Md Sharifuzzaman[1], Gauri Hasabnis[1], Sheikh Ahmed Abu Saleh[1], Leonard Siebert[2], Jan-Bernd Hövener[3], Gregor Maschkowitz[4], and Zeynep Altintas[1]**

[1]*Chair for Bioinspired Materials and Biosensor Technologies, Institute of Materials Science, Faculty of Engineering, Kiel University, Kaiserstr. 2, 24143 Kiel, Germany*

[2]*Chair for Functional Nanomaterials, Institute of Materials Science, Faculty of Engineering, Kiel University, Kaiserstr. 2, 24143 Kiel, Germany*

[3]*Section Biomedical Imaging, Molecular Imaging North Competence Center (MOIN CC), Department of Radiology and Neuroradiology, University Medical Center Schleswig-Holstein and Kiel University, 24118 Kiel, Germany*

[4]*Institute for Infection Medicine, Kiel University, Brunswiker Str. 4, 24105 Kiel, Germany*

Corresponding author: Z. Altintas

**E-mail: zeynep.altintas@tf.uni-kiel.de*

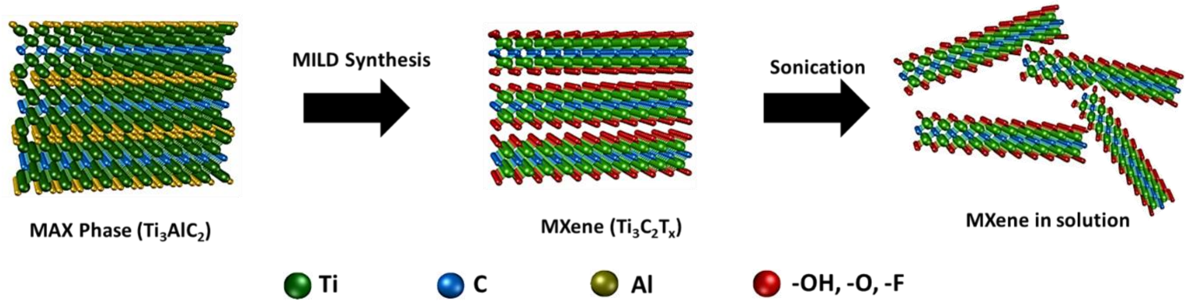

Figure S1. MXene synthesis schematic from MAX-Phase

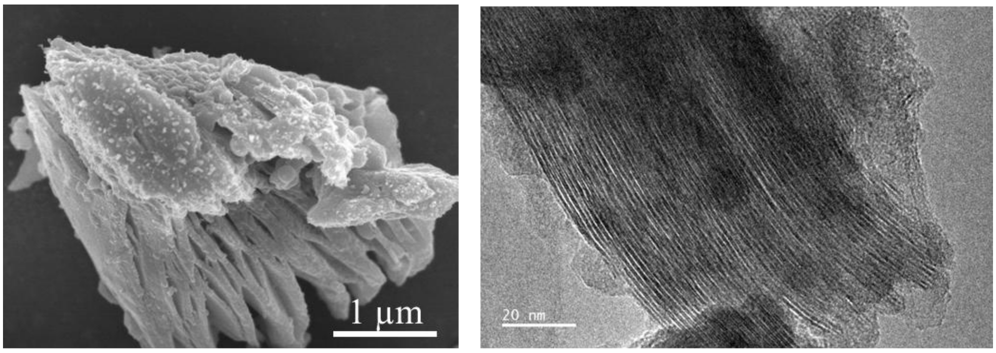

Figure S2. SEM and TEM image of accordion-like MXene sheet

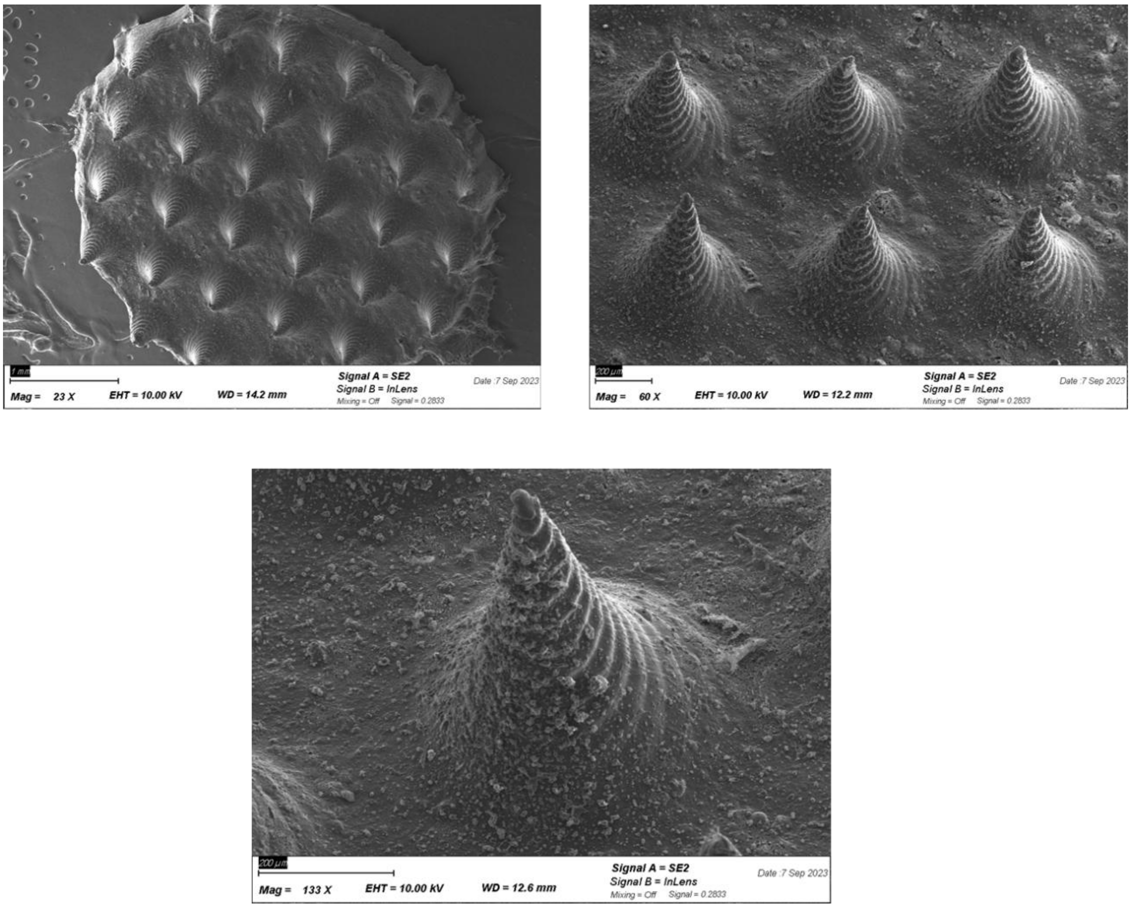

Figure S3. LSPS-based circular-cut replaceable HFMNs and their morphology.

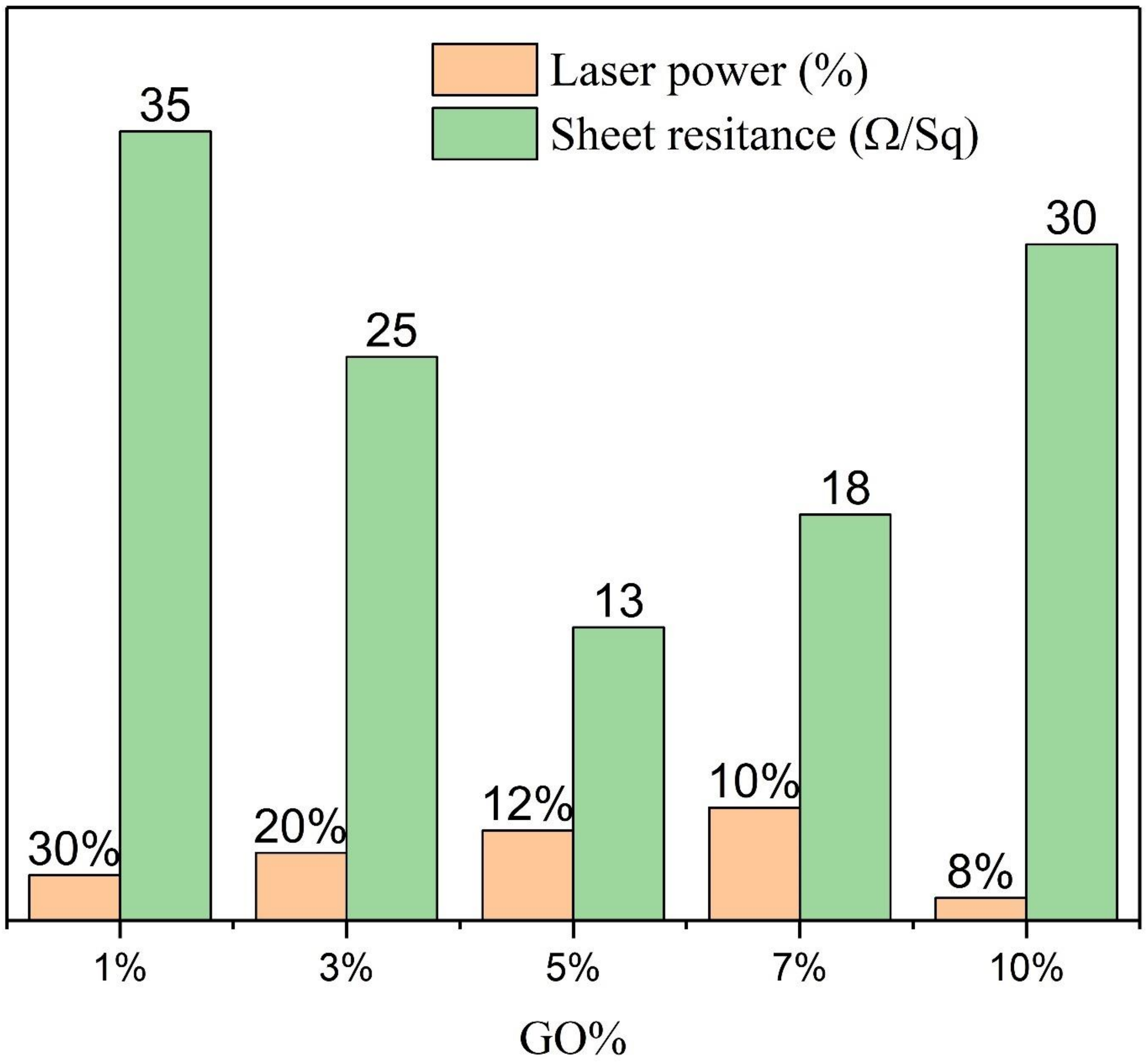

Figure S4. The sheet resistance of the hydrogel can vary based on the laser parameters. The laser power needed for the highest electrical conductivity decreased as the percentage of GO increased up to 5%.

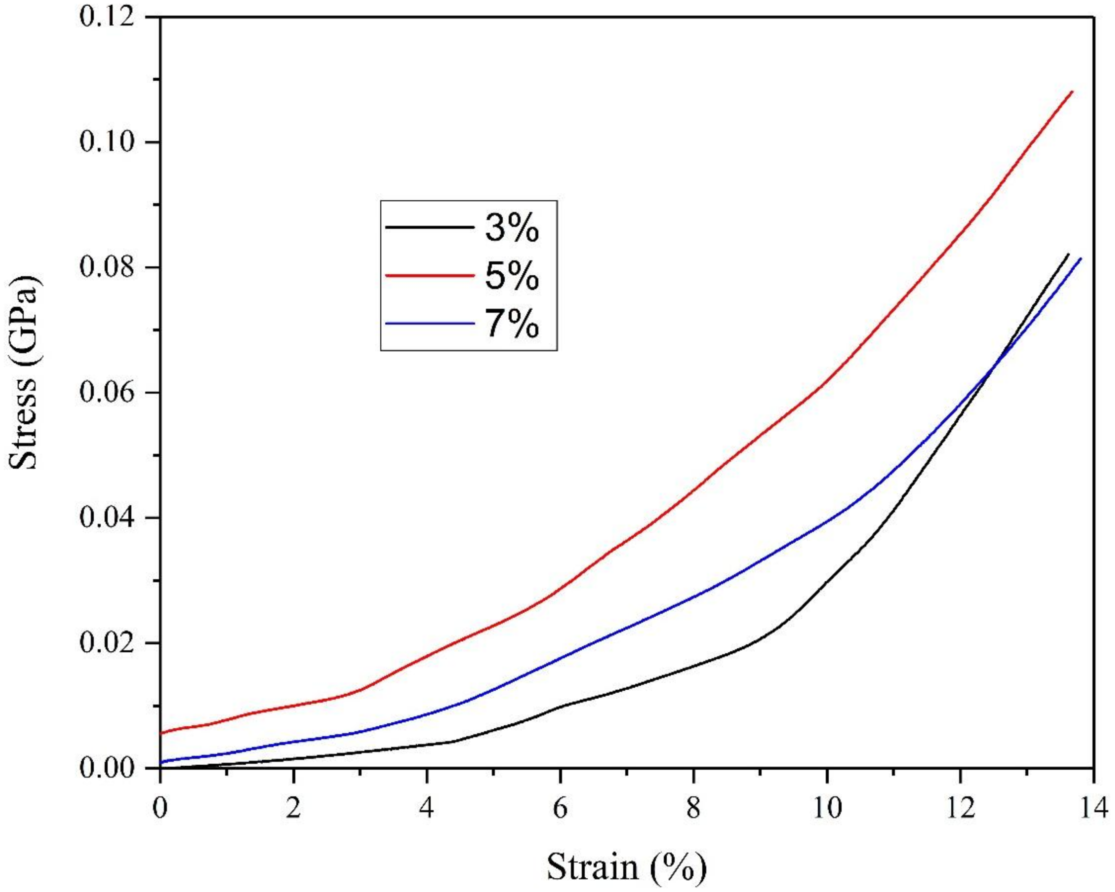

Figure S5. Nominal stress-strain curves of the microneedle arrays for a maximum applied force of 4 N.

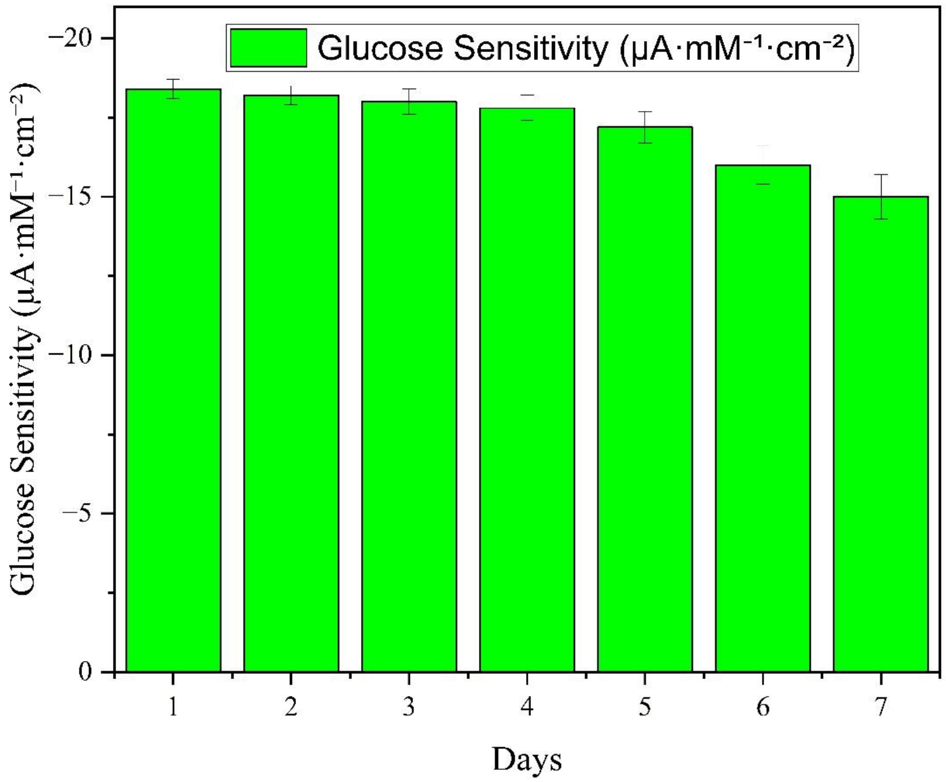

Figure S6. Stability of the Glu sensor.

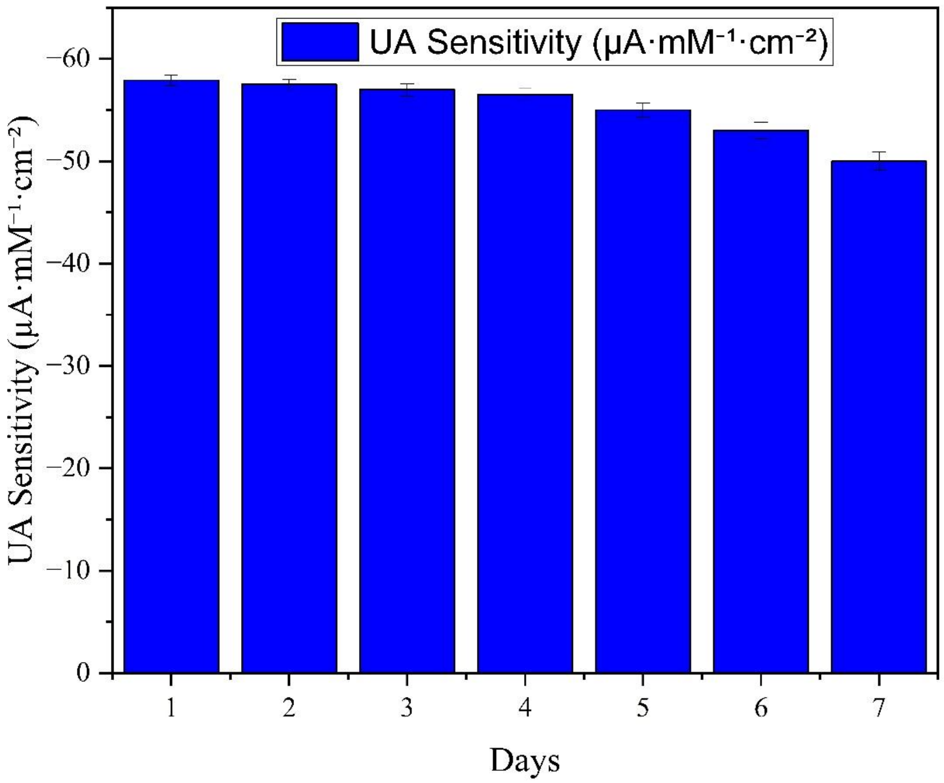

Figure S7. Stability of UA sensor.

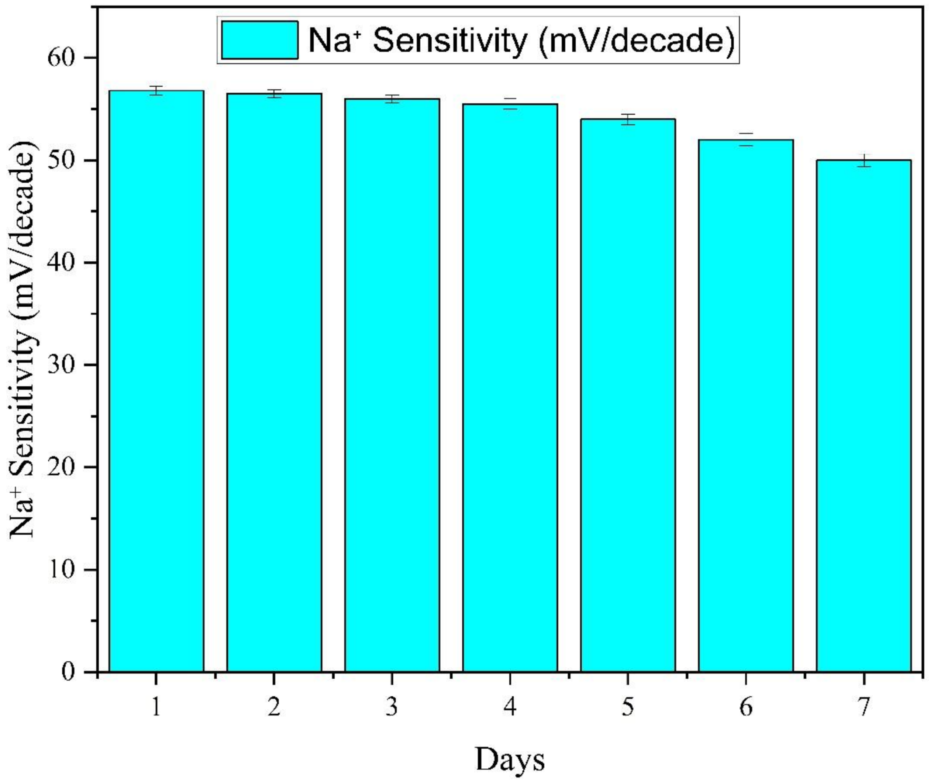

Figure S8. Stability of Na^+ sensor

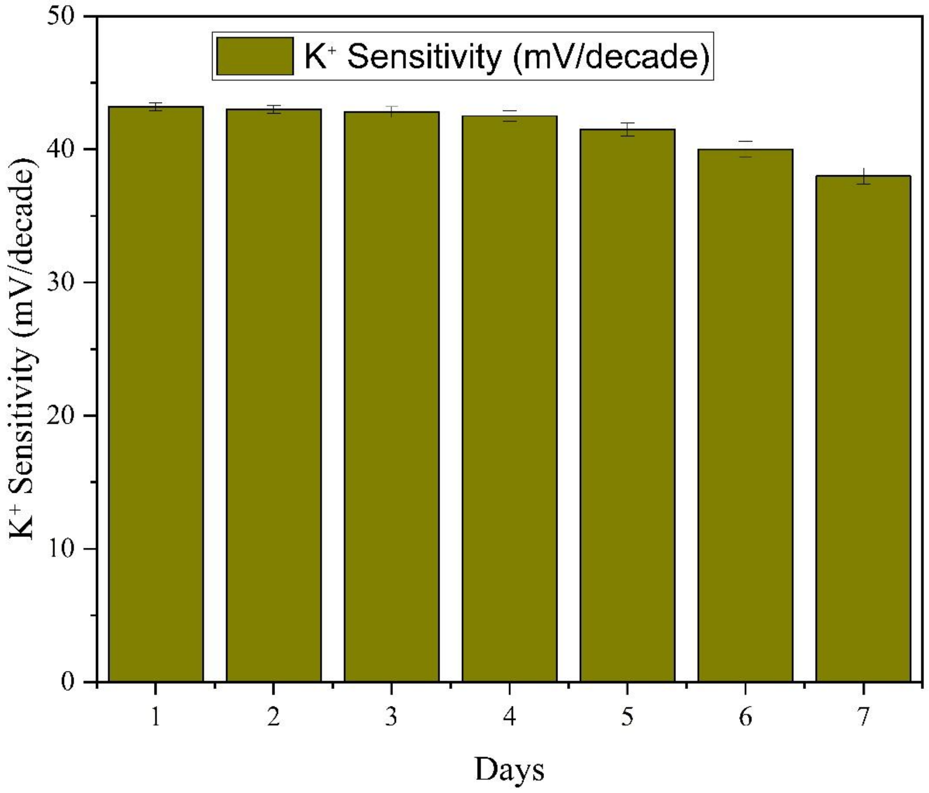

Figure S9. Stability of K^+ sensor

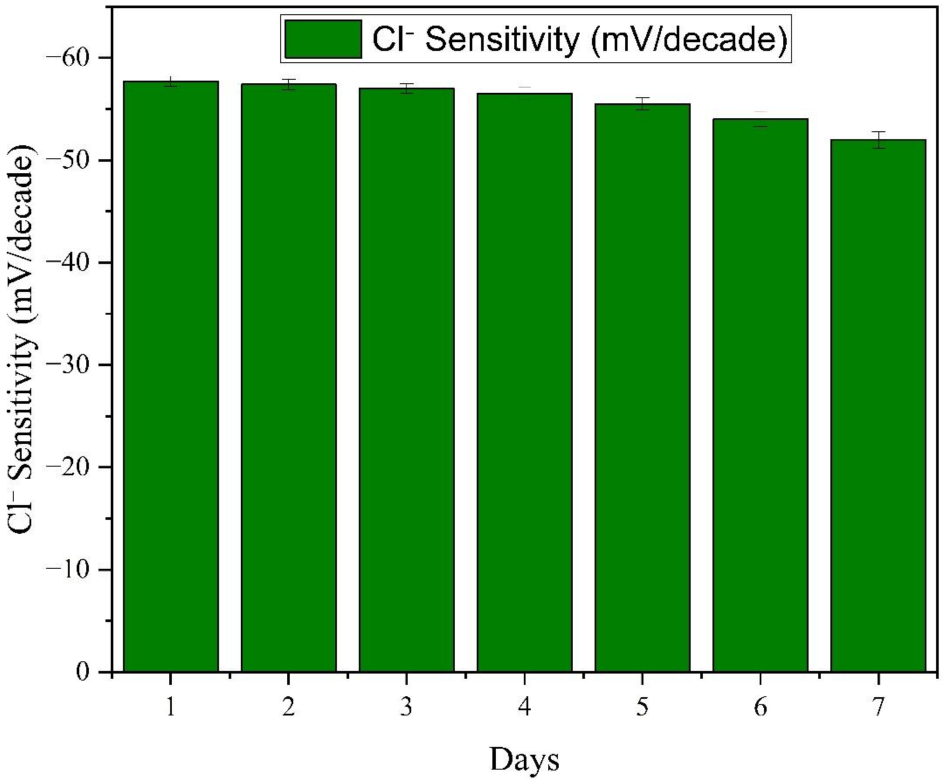

Figure S10. Stability of Cl^- sensor.

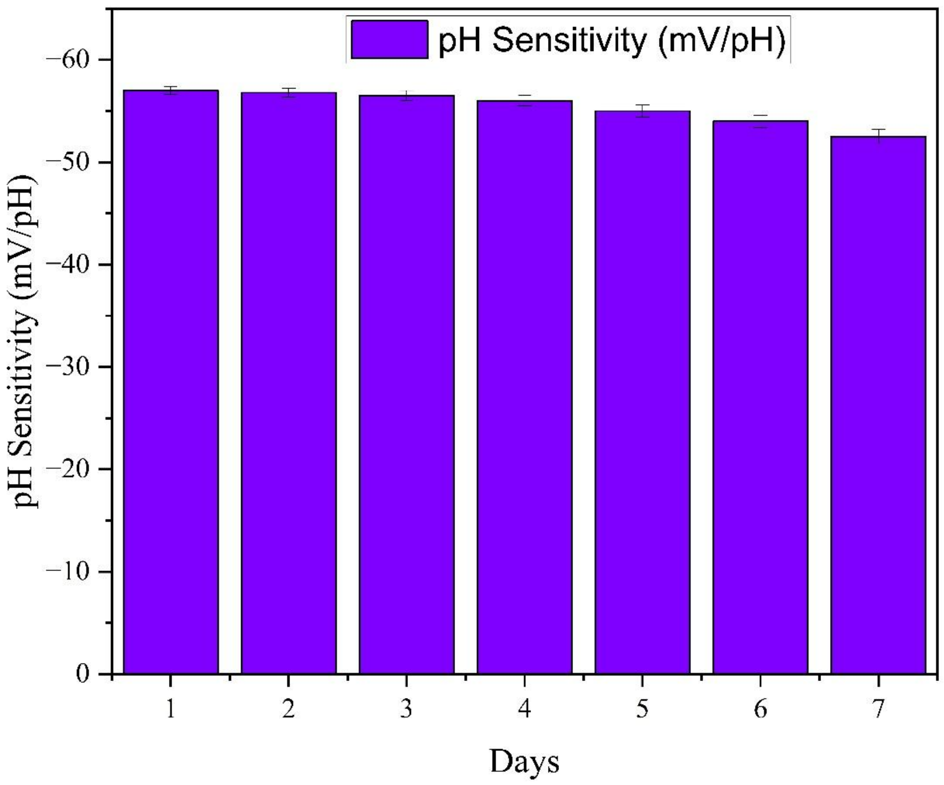

Figure S11. Stability of pH sensor.

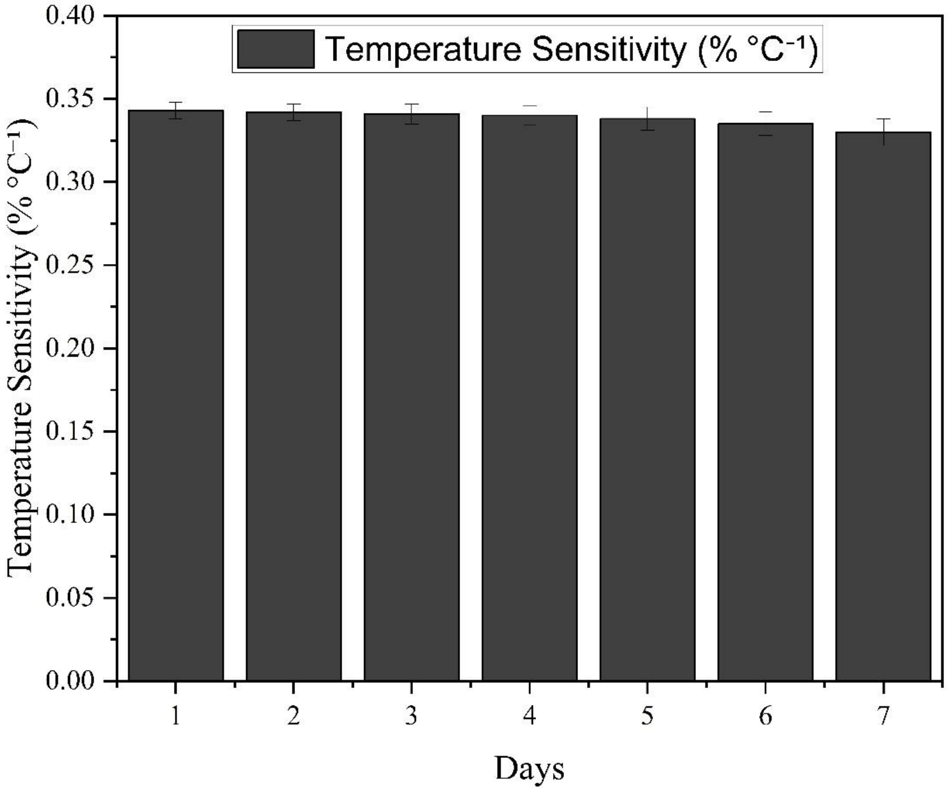

Figure S12. Stability of T sensor.

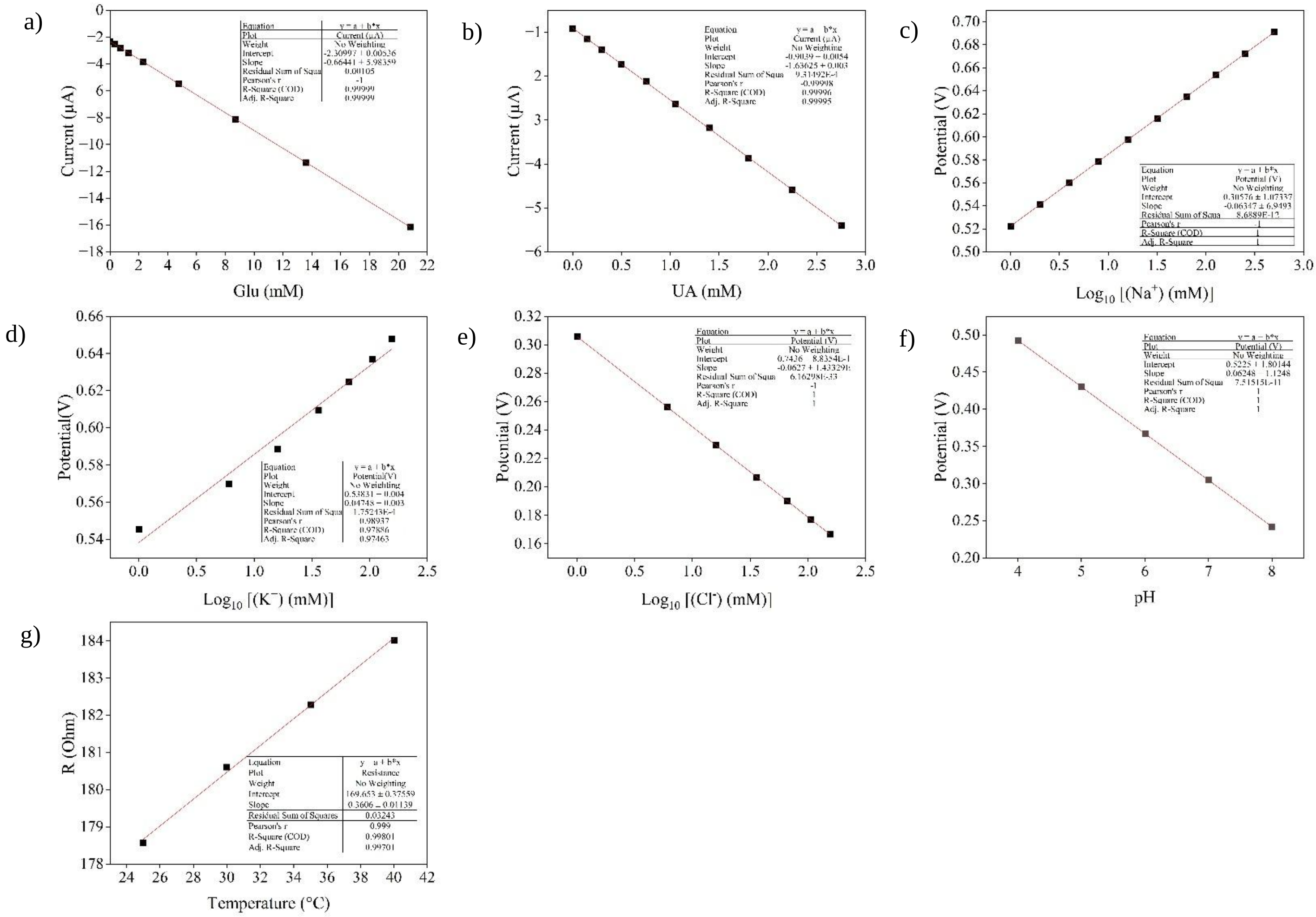

Figure S13. Characterization of the conventional wearable sensor patch used for comparative *in vivo* analysis. Calibration plots for the seven-biomarker sensor array integrated into the conventional patch, used as the benchmark for the in vivo study. All characterizations were performed in simulated interstitial wound fluid (SIWF) using a protocol analogous to that for the HFMNs sensors. The plots show: (a) Amperometric response of the glucose (Glu) sensor. (b) Amperometric response of the uric acid (UA) sensor. (c) Potentiometric response of the sodium (Na^+) sensor. (d) Potentiometric response of the potassium (K^+) sensor. (e) Potentiometric response of the chloride (Cl^-) sensor. (f) Potentiometric response of the pH sensor. (g) Resistive response of the temperature sensor. The linear fits (red lines) confirm the reliable and quantitative performance of each sensor across its respective physiological range, validating the patch as a functional benchmark.

Hydrogel Microneedle	Conductivity (S/cm)	Ref.
PEDOT:PSS-doped DA-HA	0.000346	[1]
PEDOT:PSS coated	0.0617	[2]
PEDOT:PSS/ DA-HA/AgNPs/Pt/NPs	1.65	[3]
**LSPS-based HFMNs**	**3.84**	**This work**

Table S1. Conductivity of the hydrogel-based microneedle

DA= dopamine, HA= hyaluronic acid

References

1. Odinotski, S.; Dhingra, K.; GhavamiNejad, A.; Zheng, H.; GhavamiNejad, P.; Gaouda, H.; Mohammadrezaei, D.; Poudineh, M. A Conductive Hydrogel-Based Microneedle Platform for Real-Time pH Measurement in Live Animals. *Small* 2022, *18*, 2200201, doi:10.1002/smll.202200201.
2. Keirouz, A.; Mustafa, Y.L.; Turner, J.G.; Lay, E.; Jungwirth, U.; Marken, F.; Leese, H.S. Conductive Polymer-Coated 3D Printed Microneedles: Biocompatible Platforms for Minimally Invasive Biosensing Interfaces. *Small* 2023, *19*, 2206301, doi:10.1002/smll.202206301.
3. GhavamiNejad, P.; GhavamiNejad, A.; Zheng, H.; Dhingra, K.; Samarikhalaj, M.; Poudineh, M. A Conductive Hydrogel Microneedle-Based Assay Integrating PEDOT:PSS and Ag-Pt Nanoparticles for Real-Time, Enzyme-Less, and Electrochemical Sensing of Glucose. *Adv. Healthc. Mater.* 2023, *12,* 2202362, doi:10.1002/adhm.202202362.
